# Measuring age-dependent viscoelasticity of organelles, cells and organisms with time-shared optical tweezer microrheology

**DOI:** 10.1038/s41565-024-01830-y

**Published:** 2025-01-02

**Authors:** Frederic Català-Castro, Santiago Ortiz-Vásquez, Carmen Martínez-Fernández, Fabio Pezzano, Carla Garcia-Cabau, Martín Fernández-Campo, Neus Sanfeliu-Cerdán, Senda Jiménez-Delgado, Xavier Salvatella, Verena Ruprecht, Paolo-Antonio Frigeri, Michael Krieg

**Affiliations:** 1 ICFO—Institut de Ciències Fotòniques, Castelldefels, The Barcelona Institute of Science and Technology, Barcelona, Spain; 2 Center for Genomic Regulation (CRG), The Barcelona Institute of Science and Technology, Barcelona, Spain; 3 Institute for Research in Biomedicine (IRB Barcelona), The Barcelona Institute of Science and Technology, Barcelona, Spain; 4 ICREA, Barcelona, Spain; 5 Universitat Pompeu Fabra (UPF), Barcelona, Spain; 6 Impetux Optics, Barcelona, Spain

**Keywords:** Biomaterials, Biosensors, Biological physics, Characterization and analytical techniques, Biomedical engineering

## Abstract

Quantifying the mechanical response of the biological milieu (such as the cell’s interior) and complex fluids (such as biomolecular condensates) would enable a better understanding of cellular differentiation and aging and accelerate drug discovery. Here we present time-shared optical tweezer microrheology to determine the frequency- and age-dependent viscoelastic properties of biological materials. Our approach involves splitting a single laser beam into two near-instantaneous time-shared optical traps to carry out simultaneous force and displacement measurements and quantify the mechanical properties ranging from millipascals to kilopascals across five decades of frequency. To create a practical and robust nanorheometer, we leverage both numerical and analytical models to analyse typical deviations from the ideal behaviour and offer solutions to account for these discrepancies. We demonstrate the versatility of the technique by measuring the liquid–solid phase transitions of MEC-2 stomatin and CPEB4 biomolecular condensates, and quantify the complex viscoelastic properties of intracellular compartments of zebrafish progenitor cells. In *Caenorhabditis elegans*, we uncover how mutations in the nuclear envelope proteins LMN-1 lamin A, EMR-1 emerin and LEM-2 LEMD2, which cause premature aging disorders in humans, soften the cytosol of intestinal cells during organismal age. We demonstrate that time-shared optical tweezer microrheology offers the rapid phenotyping of material properties inside cells and protein blends, which can be used for biomedical and drug-screening applications.

## Abstract (web summary)

An optical-tweezer-based nanorheometer is developed to quantify the viscoelasticity of biomolecular condensates and intracellular compartments in vivo during aging and mutations that cause nuclear envelopathies in a *Caenorhabditis elegans* model.

## Main

The many weak interactions between individual molecules in our body give rise to complex, frequency-dependent responses to self-generated and external forces. Such viscoelastic mechanics are important for many physiological and pathological processes including, among others, cell division^1^, cell migration^2^, mechanotransduction^3^ and intracellular transport^4^. Recent data indicate that alterations in the way cells and their components react to mechanical forces are linked to cancer and neurodegeneration^5,6^. In particular, many phase-separated, liquid-like condensates exhibit age-dependent changes in their mechanical properties^7,8^. Their transition from a liquid-like to a gel or glassy state has been associated with a poor prognosis of many neurodegenerative disorders^9,10^. Therefore, obtaining precise characterizations of their time-dependent microrheological properties could yield valuable insights for drug development and diagnosis.

Many techniques have been put forward that afford the characterization of the cell’s rheological properties, including atomic force microscopy, Brillouin spectroscopy and genetically encoded reporters for stress and strain^11^. Yet, none of them can simultaneously exert forces and measure their resulting effects on cell mechanics and mechanotransduction inside living systems.

Optical tweezers are particularly well suited to derive the material properties of biological materials as they operate in the piconewton range, characteristic for molecular interactions, and can measure the position of micrometre-sized objects with sub-nanometre accuracy in three dimensions using non-invasive infrared laser light^12^. The ability to measure in small volumes also permits the analysis of the heterogeneity of the response, rather than providing a bulk modulus from macroscopic measurements—a key point for experiments aimed at understanding subcellular mechanical compartmentalization. Optical-tweezer-based active microrheology is a powerful technique for probing the mechanical properties of complex fluids in biological systems, such as the cytoplasm of living cells and cancer spheroids and in living animals and biomolecular condensates (BMCs)^12,13^. This method involves measuring the motion of small particles suspended within a fluid (or embedded in a viscoelastic gel or the cell cytoplasm), due to an oscillating optical trap^14,15^. The frequency-dependent probe displacement ${\hat{x}_{{\rm{p}}}}(\omega )$ in response to the active force $\hat{F}\,(\omega )$ captures the elasticity and viscosity of the material over the observed rheological spectrum.

However, measuring the mechanical and rheological properties requires the simultaneous recording of stress and strain. Therefore, using optical tweezers requires two laser beams to measure the optical force acting onto an optically trapped probe and its displacement resulting from such force^1,16,17^. In this paper, we present an active microrheology method using a single time-shared laser to create two optical traps—one for driving active oscillations and the other for static displacement detection. We developed theory and experiments to study the rheological properties of BMCs, cells and animals, focusing on zebrafish and *Caenorhabditis elegans* to explore the relationship between material properties, morphogenesis, aging and disease.

## Implementation of TimSOM

**Fig. 1:**
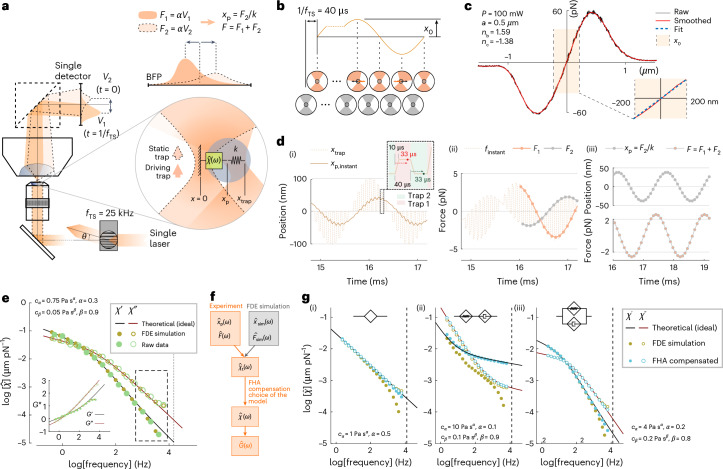
TimSOM. **a**, Schematic of TimSOM with direct light momentum sensing of optical forces. A single laser beam is time shared at 25 kHz between a driving (1) and a static detection (2) trap. The driving trap (orange) oscillates around the trapped particle, whereas the static trap (light orange, dashed line) monitors the particle position as *x*_p_ = *F*_2_/*k*. For clarity, only the spring for the driving trap was indicated, noting that both traps have the same spring constant. The optical force acting onto the probe particle corresponds to the addition of forces exerted by the two traps: *F* = *F*_1_ + *F*_2_, which are obtained as *F*_1,2_ = *αV*_1,2_, where *α* is the volt-to-piconewton conversion factor of a single, direct light momentum force sensor. **b**, Time-sharing position and force measurement sequence. Although trap 2 remains motionless at the optical axis to detect bead displacements through the BFP interferometry, trap 1 applies an active sinusoidal perturbation with amplitude A at the time-sharing frequency *f*_TS_. The schematic on the bottom represents the deflection of the laser beam by the trapped particle for the driving (orange) and static (grey) traps. The orange (black) arrow indicates the optical (material) force acting onto the bead for the driving trap. **c**, Force profile acquired by sweeping the trap across a 1 µm polystyrene microsphere embedded in the cytoplasm of a zebrafish cell. The shaded area indicates that the force is linear with displacement over the amplitude of the rheology routine. The blue dotted line is *F* = *kx*. **d**–**g**, Quantitative description of bead motion in TimSOM. Simulation of the instantaneous position (i) of the probe particle in water using the FDE method (*A* = 100 nm; *f* = 625 Hz; *k* = 50 pN µm^–1^; water viscosity, *η* = 10^−3^ Pa s) and the resulting instantaneous optical force acting onto the probe (ii, dashed line) (**d**). Interleaved force values for the static and driving traps, sampled at *f*_TS_/2 = 12.5 kHz with a delay of 33 µs (Supplementary Text 2), are indicated as the orange and grey circles, respectively. The inset in (i) shows the time-sharing properties of the driving (trap 1) and static (trap 2) traps with the rise time of 10 µs. In (iii), the probe position and total force are shown. Response function derived from the FDE simulation (orange circles) with the parameters indicated in the top left and experimental data acquired in a zebrafish progenitor cell (green circles) using the time-shared microrheology routine (**e**). The solid lines show the expected, ideal behaviour of a fractional Kelvin–Voigt material. The inset shows the complex shear modulus. The dashed box indicates high frequencies with expected deviations due to the non-simultaneous measurement of stress and strain. Analytical pipeline to retrieve *G* modulus from the deviated measurements and/or FDE simulations (**f**). Response function (*χ*′, storage; *χ*″, loss) of the ideal scenario (theoretical), the time-shared simulations (FDE) and the compensated data points (FHA) for a single springpot (i), fractional Maxwell model (ii) and fractional Kelvin–Voigt (iii) model (**g**). The parameters used for the simulation are indicated in each panel and the legend is indicated on the right. Source data

To conceptualize a simplified microrheology experiment, we consider a single laser source to drive the probe and measure the resulting displacement. The laser is time shared and generates two traps that alternate between two positions. Thus, time sharing generates a discontinuous but quasi-simultaneous stress/strain measurement (Fig. 1a,b). The advantage is reduced complexity and alignment, and, as a consequence of the shared laser source, the traps have identical power, position sensitivity *β*_1_ = *β*_2_ ≡ *β* and trapping stiffness *k*_1_ = *k*_2_ ≡ *k/* 2, where *k* is the sum of the stiffness of the two traps (Fig. 1a and Supplementary Text 1.2).

We envision that at the beginning of the routine, both traps are centred on the microsphere (Fig. 1b). Trap 1, the driving trap, starts to oscillate at a given frequency *ω* and amplitude *A*, that is, *x*_1_(*t*) = *A*sin(*ωt*). Trap 2, the static trap, remains fixed at *x*_2_(*t*) = 0 (Fig. 1b). The amplitude must be in the linear range of the trap (*A* = 200 nm; Fig. 1c) and, therefore, smaller than the radius of the microsphere (*a* = 0.5 µm). Thus, the probe particle will feel a force from both traps. Because the traps are of equal strength, we expect that the static laser exerts a force on the probe back into the starting position. Depending on the viscoelastic properties of the surrounding material, the measured microsphere trajectory may deviate from the ideal case (Supplementary Text 1.4).

To model and understand the effects of time sharing, we first simulated the displacement of the particle under the influence of the two intermittent traps, solving the equation of motion under an external force in a viscoelastic matrix best described by power laws^18^. In particular, we consider the fractional Kelvin–Voigt or Maxwell models^19^. These generalized viscoelastic models extend the applicability of the classical Kelvin–Voigt, Maxwell and structural damping models. They have been used to describe the power-law behaviour of many biological samples and gels^19^, including the intracellular cytoplasm^1,20,21^. First, we numerically computed the probe trajectory using the fractional derivative equations (FDEs) that describe motion in materials with power-law rheology (Supplementary Text 1.5). We found that each trap influences the instantaneous probe trajectory *x*_p,instant_(*t*), leading to a sawtooth shape (Fig. 1d). For a viscoelastic material, this deviation may depend on the rate at which it is deformed and, hence, the time-sharing frequency. We are interested in how this generally affects the response obtained from the time-shared optical tweezer microrheology (TimSOM) measurement in viscoelastic media.

For viscoelastic materials, the response $\hat{\chi}$ of the microsphere, as it transitions from the ‘current’ to the ‘new’ position under force, depends on the stimulation frequency (*ω*). This is described with the complex response function $\hat{\chi}(\omega)$ and the complex shear modulus $\hat{G}(\omega)$, which consist of a real part and an imaginary part, typically denoted as $\hat{G}={G}^{{\prime}}$+i*G*″. *G*′ is the storage modulus and *G*″ is the loss modulus (Supplementary Text 1.2 provides the derivation), which describe the material’s elastic and viscous behaviours, respectively.

1a $$\hat{\chi }\left(\omega \right)=\frac{\hat{x}\left(\omega \right)}{{\hat{F}}_{\text{tot}}\left(\omega \right)}=-\frac{2{\hat{V}}_{2}\left(\omega \right)}{k\left[{\hat{V}}_{1}\left(\omega \right)+{\hat{V}}_{2}\left(\omega \right)\right]},$$

1b $$\hat{G}\left(\omega \right)=\frac{1}{6\uppi a}\cdot\frac{1}{\hat{\chi }(\omega )}=-\frac{k}{12\uppi a}\cdot\frac{{\hat{V}}_{1}(\omega )+{\hat{V}}_{2}(\omega )}{{\hat{V}}_{2}(\omega )},$$

where *V*_1_ and *V*_2_ are the voltage signals of a position-sensitive detector placed at the back-focal plane (BFP) of a direct force measurement sensor^22^. We can now use equations (1a) and (1b) to calculate the time-sharing response of the microsphere as it moves within a viscoelastic solid, over frequencies ranging from 0.1 Hz up to the Nyquist frequency (6.25 kHz). Intuitively, we indeed found a deviation from the ideal response function at high frequencies (Fig. 1e, beige points versus solid line). At this stage, we directly incorporated TimSOM into our optical tweezer setup with fast-scanning acousto-optic deflectors (AODs) and direct light momentum force detection^23^ to evaluate this prediction. Clearly, when we performed a rheology routine in the viscoelastic cytoplasm of a living cell, we observed the same deviation from the ideal fractional Kelvin–Voigt behaviour, as predicted by the FDE simulation (Fig. 1e).

Next, to understand and eventually correct for this deviation, we obtained an analytical solution of the probe motion—and the resulting $\hat{\chi}_t(\omega)$ as the linear response function of the time-sharing method—through a first-harmonic approximation (FHA) of the trap-positioning sequence in the frequency domain (Supplementary Text 2 shows the derivation). Our analytical solution allows us not only to predict the time-sharing response function based on the ideal, time-continuous response function but also to retrieve the artefact-free response function *χ*(*ω*) from the deviated measurement *χ*_t_(*ω*) (Fig. 1f,g; for details, see Supplementary Texts 1.8 and 1.12).

To demonstrate that the compensation can be applied generally and is, indeed, able to retrieve the ideal, artefact-free response function (Fig. 1g, solid lines), we first simulated the trajectories and the response function using the FDEs in various materials, such as power law and the fractional Maxwell and Kelvin–Voigt materials (Fig. 1g (beige points) and Supplementary Text 1.11). For viscoelastic liquids (for example, Maxwell materials), the deviations strongly affect the real part of the response function, such that the solid behaviour at frequencies larger than the crossover frequency is not accessed for inadequately stiff traps. The bead jumps instantaneously between the two traps, which creates a deviation from the expected viscoelastic behaviour in the high-frequency range of materials in which the elastic contributions dominate. Strikingly, when we applied the compensation algorithm (Supplementary Equation (46)), we recovered an almost perfect match (Fig. 1g, blue points) to the ideal behaviour.

## TimSOM measures 0.1 Pa–100 kPa over 5 decades of frequencies

**Fig. 2:**
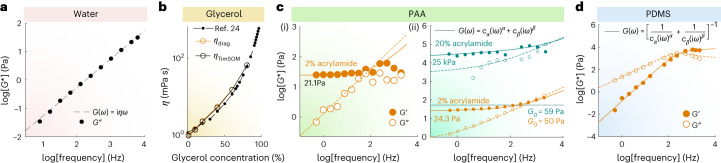
TimSOM correctly measures known viscoelastic materials. **a**, Representative experimental data of the G* modulus for water obtained from TimSOM compensated using Supplementary Equation (46). The dashed line indicates the fit to the data. **b**, Viscosity of different glycerol mixtures extracted from TimSOM (orange circles) and the classical drag force method (black circle). Viscosity was obtained from a linear fit to the averaged force values obtained for a series of triangles with different velocities (Extended Data Fig. 2a). The closed circles are the known references taken from ref. ^24^. **c**, Rheological spectra of different PAA gels. Real (solid circles) and imaginary part (open circles) of the *G* modulus derived from the creep compliance force-clamp measurement $(J(t)\rightarrow\hat{G}(\omega))$ on a 2% PAA gel (i). The solid or dashed line represents a fit of the data to the Fractional Kelvin–Voigt model with a low-frequency elastic modulus of *C*_*α*_ = 21.1 ± 8.83 Pa (mean ± confidence interval of 95%). TimSOM measurements on two different gels with varying stiffness values (ii). The orange gel is the exact same one as that in panel (i). The filled (open) circles correspond to the real (imaginary) part. The solid (dashed) lines are the real (imaginary) part of the fractional Kelvin–Voigt model, as specified in the legends. For the PAA gel, using the TimSOM method, we get *C*_*α*_ = 24.3 ± 1.28 Pa (confidence interval of 95%). The mint-coloured dots and lines correspond to a 20% acrylamide gel. The modulus is indicated in the figure, together with the trap stiffness *G*_0_ that was used to measure each gel. **d**, Representative raw data of the *G* modulus for PDMS obtained through equations (1a) and (1b), compensated using the FHA method (Supplementary Equation (46)). The filled (open) circles correspond to the real (imaginary) part. The solid (dashed) lines are the real (imaginary) part of the model specified in the legends. Source data

We then tested the TimSOM scheme on three different materials with known rheological properties. The viscosity of water (Fig. 2a) and the different glycerol mixtures (Fig. 2b) extracted from TimSOM were consistent with the values measured from classical drag force measurements (Fig. 2b and Extended Data Fig. 2a) and the published literature^24^. Second, we fabricated different polyacrylamide (PAA) gels ranging from 20 Pa to 100 kPa (Methods). Specifically, when we compared the same soft gels using creep compliance measurements^25^ (Fig. 2c(i) and Extended Data Fig. 2b) and TimSOM (Fig. 2c(ii)), we found similar values for the frequency-dependent shear modulus that closely aligned with the fractional Kelvin–Voigt model (Fig. 2c). For the stiffest gels (20% acrylamide), we measured a low-frequency plateau modulus of ~30 kPa that reached 100 kPa at short timescales (Fig. 2c(ii)). Importantly, even for these stiff gels, the frequency-domain passive peaks were at least one standard deviation higher than the noise baseline, demonstrating the excellent force and displacement sensitivity of TimSOM (Extended Data Fig. 2c(ii)).

Last, we also tested TimSOM on microspheres embedded in freshly prepared and uncured 10:0.1 polydimethylsiloxane (PDMS) prepolymer/curing agent mixtures (Fig. 2d). Consistent with previous reports, pregelation PDMS was best described as a fractional Maxwell viscoelastic gel^19^ with a plateau modulus at high frequencies of ~50 kPa (refs. ^26,27^). Taken together, TimSOM retrieved viscoelastic moduli over five orders of magnitude across a range from 0.1 Hz to 6.25 kHz.

## Probing the viscoelasticity of aging protein condensates

**Fig. 3:**
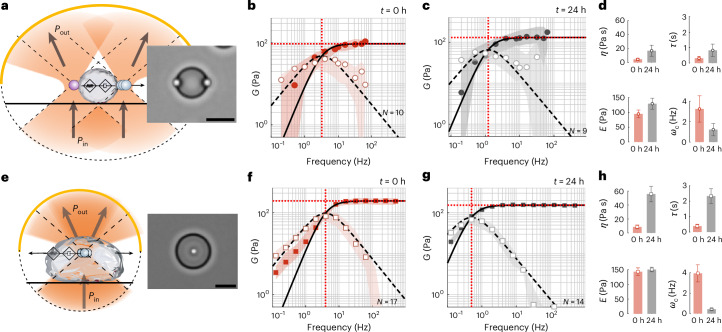
Viscoelastic properties of BMCs. **a**–**d**, Schematic (**a**) and snapshot of an MEC-2/UNC-89 protein droplet measured with a pair of optically trapped polyethylene-glycol-terminated microspheres in a dual optical trap. Scale bar, 10 µm. *P*_in_ and *P*_out_ define the light momentum before and after interacting with the trapped microsphere (Supplementary Video 1). **b**,**c**, Storage (*G*′(*ω*), filled circles) and loss (*G*″(*ω*), open circles) moduli measured in the dual optical trap at *t* = 0 h (**b**) and *t* = 24 h (**c**) after condensate formation. The solid and dashed lines are the real and imaginary parts of the *G* modulus, derived from a fit of the Maxwell model to the acquired data. The circles and shadows are the median and ±25% quantiles measured for *N* = 10 (**b**) and *N* = 9 (**c**) droplets. **d**, Variation in the fitting parameters showing the changes in dynamic viscosity *η* (Pa s), stiffness *E* (Pa), time constant *τ* = *η*/*E* (s) and crossover frequency *ω*_c_ = 1/*τ* (Hz) over 24 h condensate maturation in the dual optical trap. The mean and standard deviation derived from the fits in **b** and **c**. **e**–**h**, Schematic (**e**) and snapshot of a TimSOM experiment on MEC-2/UNC-89 protein droplet with an embedded carboxylated microbead (Supplementary Video 2). **f**,**g**, Storage (*G*′(*ω*), filled squares) and loss (*G*″(*ω*), open squares) moduli measured with TimSOM at *t* = 0 h (**f**) and *t* = 24 h (**g**) after condensate formation. The solid and dashed lines are the real and imaginary parts of the G modulus, derived from a fit of the Maxwell model to the acquired data. The squares and shadows are the median and ±25% quantiles measured for *N* = 17 (**f**) and *N* = 14 (**g**) droplets. **h**, Variation in the fitting parameters (dynamic viscosity *η* (Pa s), stiffness *E* (Pa), time constant *τ* = *η*/*E* (s) and crossover frequency *ωc* = 1/*τ* (Hz)) over 24 h extracted from the TimSOM routine. The mean and standard deviation derived from the fits in **f** and **g**. Source data

BMCs, play a crucial role in cytoplasmic organization, and their change in the material state has consequences for health and disease. Recently, laser tweezer microrheology revealed that many BMCs display an age-dependent change in viscosity^7,8^. These experiments are commonly performed in a dual optical trap, in which a droplet is sandwiched between two trapped, active and passive microspheres (Fig. 3a–d and Supplementary Video 1). The complex shear modulus and surface tension of the material are then extracted from the active force measurement and passive bead displacement, with the assumption that the microsphere radius is considerably smaller than the protein droplet and that the viscosity of the dilute phase is known^7,28^. To relax these assumptions and simplify the experiment with a single trap, we performed TimSOM on MEC-2 BMCs as a model for an age-dependent maturation process and compared these results with the dual-trap assay (Fig. 3e–h, Supplementary Video 2 and ref. ^8^). After applying the compensation routines for a fractional Maxwell material, we found a similar frequency response in both configurations, and a substantial shift in the crossover frequency to lower values after 24 h of droplet formation (Fig. 3d, h). Interestingly, the values for stiffness and viscosity were slightly higher when measured in the centre of the droplet compared with those taken near the interface. To test if TimSOM can be applied to other BMCs, we tested BMCs composed of cytoplasmic polyadenylation element binding protein 4 (CPEB4), an RNA-binding protein that regulates translation through cytoplasmic changes in poly(A) tail length, with links to idiopathic autism spectrum disorder^29^ and with previously uncharacterized shear modulus^30^. In their ‘naive’ phase (within 2 h after droplet formation in vitro), the mechanical response function confirmed their fractional Maxwell behaviour (Extended Data Fig. 3a), and the BMCs displayed an ~10× slower relaxation timescale and increased viscosity (Extended Data Fig. 3b) compared with MEC-2/UNC-89. Moreover, we found that the CPEB4 condensates stiffen very quickly after formation, visible by the appearance of fibres (Extended Data Fig. 3c), which prevented the rheological characterization of later time points.

## Rheological fingerprint of intracellular organelles

**Fig. 4:**
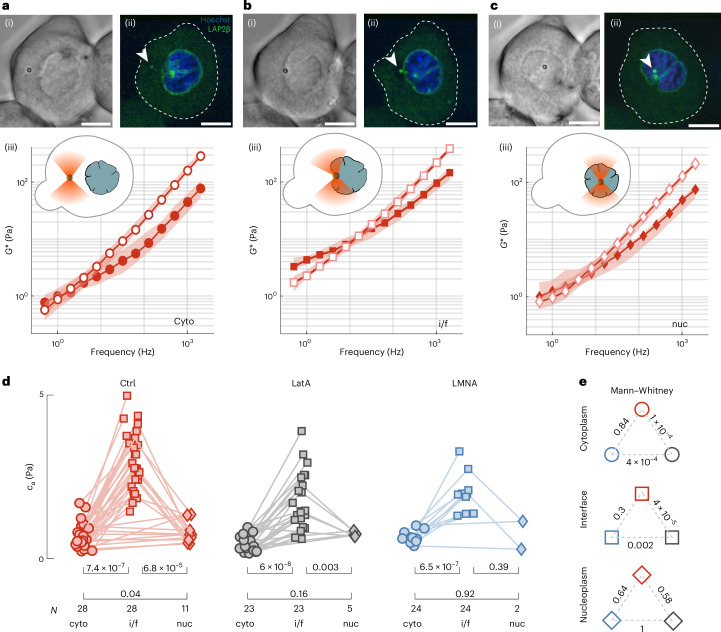
Cytoplasm versus nuclear rheology. **a**–**c**, Representative bright-field (i) and confocal (ii) images of a zebrafish progenitor cell stained with Hoechst (blue) to label the nucleus and expressing Lap2*β*-GFP (green) with a microsphere in its cytoplasm (cyto) (**a**), nuclear interface (i/f) (**b**) and inside the nucleus (nuc) (**c**). Frequency spectrum of the complex *G* modulus, indicating the storage (closed symbols) and loss (open symbols) moduli of the three corresponding compartments (iii). Scale bars, 10 µm. Supplementary Video 3 shows the complete routine. **d**, Stiffness (*C*_*α*_) of the cytoplasm, nuclear interface and nucleoplasm for controlm F-actin depolymerization (LatA) and LMNA overexpression conditions as extracted from the fit of a fractional Kelvin–Voigt model to the rheological spectrum. The lines connect paired data points that were acquired in the same cell with the same microsphere. For control cells, the experiments were independently repeated *n* = 9, 9 and 3 times for cyto, i/f and nuc, respectively. For LatA, *n* = 7, 7 and 3 and for lamin A, *n* = 4, 4 and 1, respectively. *P* values above the brackets derived from a paired *t*-test. Extended Data Fig. 5 and Supplementary Table 1 show a comparison of all the other fit parameters and their *P* values. *N* is the number of cells used in the measurement. **e**, *P* values of the indicated pairwise comparison using a two-sided Mann–Whitney *U*-test for *C*_*α*_ of the cytoplasm, nuclear interface and nucleoplasm in control, LatA treatment and lamin A (LMNA) overexpression. Source data

TimSOM facilitates intracellular rheology because direct, momentum-based optical force measurements have their greatest potential inside living cells, where traditional mechanics measurements are limited by time-consuming and sample-variant in situ calibrations^12,31^. To demonstrate TimSOM inside cells, we introduced microspheres into individual zebrafish embryos and extracted cells 4 hours post-fertilization^23^. We trapped individual microspheres and measured the complex shear modulus of the cytoplasm and at the nuclear interface. During these measurements, no history effect or nonlinear mechanical response for the duration of the rheology routine was observed (for example, heating related to laser power; Extended Data Fig. 4). The frequency-dependent *G* modulus revealed a viscoelastic response with an elastic plateau at low frequencies (Fig. 4a) and a viscous response dominated at high frequencies. Similar to other cells investigated before^20^^,21^, this response was well described by the fractional Kelvin–Voigt model (Supplementary Text 3), but much softer. The crossover frequency at which the dissipative forces dominate was ~5 Hz and above, and the cytoplasm fluidified as indicated by exponent *β* approaching 1 (Extended Data Fig. 5c). We found that the cytoplasm is stabilized by a synergistic effect of the F-actin and microtubule (MT) cytoskeleton: pharmacological perturbation of actin and tubulin polymerization leads to decreases in *C*_*α*_ and *C*_*β*_, indicating a reduction in the magnitude of the elastic and viscous moduli^21^. Both elements, however, have a different contribution to the low-frequency response. Interestingly, depolymerization of the MT cytoskeleton decreases the low-frequency power-law exponent *α*, different from what was observed in dividing tissue culture cells^1^, which points towards a partial solidification of the cytoplasm. This may be an indirect effect of MTs on the F-actin cytoskeleton, as it is known that RhoGEF is bound to the MT lattice, which gets released upon MT depolymerization to exert its effect on actin dynamics by increasing the GTPase activity^32^. Indeed, upon F-actin depolymerization, the effect of nocodazole on *α* is reversed and the cytoplasm is again more liquid like (Extended Data Fig. 6a). Taken together, G* was lower for all the frequencies after the depolymerization of F-actin (Fig. 3a and Extended Data Fig. 5d) and MT cytoskeleton (Extended Data Fig. 6a) but not after disrupting the myosin II motor activity (Extended Data Fig. 6b), indicating that the cytoskeletal network integrity rather than contractility had a major effect on the rheological signature of the cytoplasm.

Surprisingly, we found that the cytoplasm was soft enough to reposition the same microsphere to the cytoplasmic–nuclear interface, where we repeated the measurement to test the mechanical properties of the largest cellular organelle (Supplementary Video 3). In agreement with previous reports^33,34^, we found that the nuclear interface is stiffer than the cytoplasm (Fig. 4). To unravel the molecules responsible for determining G* of the interface, we conducted experiments on cells that overexpress lamin A, a key protein found in the nuclear envelope. In parallel, we also treated cells with latrunculin A (LatA) to perturb the F-actin network. The G* value of the nuclear interface in lamin A-overexpressing cells was indistinguishable from the control cells (Extended Data Fig. 5e), which do not express high levels of lamin A^35^. We also performed a creep compliance test to measure the resistance of the nucleus to large deformations (Extended Data Fig. 7a–d). Even though the nuclear envelope was more resistant on short and long timescales, its initial stiffness was unaffected by lamin A overexpression (Extended Data Fig. 7f–j). Actin around the nucleus was previously known to dampen and transmit mechanical forces^36^ and was observed in actin stainings of isolated gastrulating zebrafish progenitor cells (Extended Data Fig. 8a,b and ref. ^37^). We, thus, speculated that actin has a noticeable effect on the mechanics of the nuclear interface. Indeed, cells immediately ceased blebbing in the presence of 0.5 µM LatA^38^, with a significant reduction in the initial stiffness and resistance to deformation when we performed the large-strain creep compliance test (Extended Data Fig. 7e–j). Moreover, G* of the nuclear interface was significantly reduced in the presence of LatA compared with the control cells. However, even without a functional F-actin network, it remained significantly higher than that of the cytoplasm in the presence of LatA (Fig. 4d,e and Extended Data Fig. 5d). As the nuclear envelope originates from the endoplasmatic reticulum (ER), we asked whether or not our measurements at the nuclear interface are affected by the properties of the ER. We first stained the ER and the nucleus and found that the microsphere is indeed in close contact with the nuclear envelope, without large accumulations of ER in between (Extended Data Fig. 8c,d). We then induced morphological and mechanical changes at the ER using brefeldin A^39^. However, compared with the actin cytoskeleton, the ER had little influence on our measurements (Extended Data Fig. 8e). Together, these data show that F-actin increases G* of the nuclear–cytoplasmic interface, which is unaffected by lamin A expression at the inner nuclear membrane.

To better understand the rheological properties of the nucleus itself, we mechanically inserted microspheres into the nucleoplasm (Extended Data Fig. 7). Despite the relatively large shear moduli of the nuclear interface compared with the cytoplasm (Fig. 4a,b), the envelope was very flexible and deformed easily under modest forces exerted by the optical trap. Under a constant force of 100–150 pN onto the nuclear envelope, the microsphere entered into the nucleus, which afforded the possibility to independently measure the rheology of the nucleoplasm (Fig. 4c and Supplementary Video 4). Importantly, we thoroughly tested that the nuclei retained their integrity and functionality after microsphere insertion (Supplementary Videos 5–7 and Supplementary Text 3.2). Our rheology measurement of the nucleoplasm revealed a comparably soft material similar to the cytoplasm (Fig. 4c). The complex shear moduli, however, did not change in the presence of LatA and the expression of lamin A (Extended Data Fig. 5), indicating that the mechanical properties of the nucleoplasm are not influenced by the actin cytoskeleton and the nuclear envelope.

## Aging and nuclear envelopathies affect cytoplasmic viscoelasticity

**Fig. 5:**
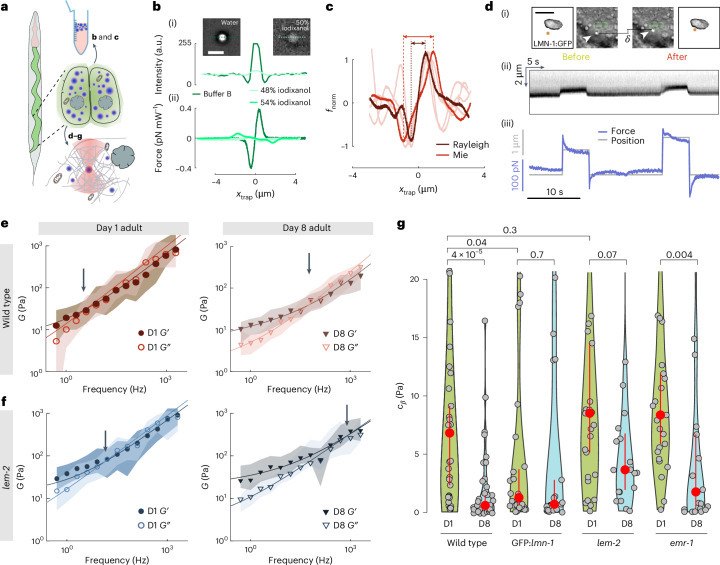
Longitudinal tissue microrheology in vivo*.* **a**, Sketch of an animal with the intestinal tissue highlighted in green and the pharynx in red. The close-up sketch shows a pair of posterior intestinal cells with lipid droplets in blue. The lipid droplets were isolated from adult animals, purified and tested under various conditions for their suitability as optical tweezer probes (**b** and **c**; Methods). For in vivo application, individual droplets were trapped to measure the rheological response of the material in its vicinity (**d**–**g**). **b**, Refractive-index matching with varying concentrations of iodixanol. Bright-field micrograph of a lipid droplet in buffer B on the left (Methods; representative for *N* = 6 droplets) and in 48% of iodixanol (right; *N* = 12) (i). The graph shows the intensity profile along the dotted line indicated in the photograph. Scale bar, 2 µm. Force profile on a droplet in the matched conditions, for 0%, 48% and 54% of iodixanol (ii). **c**, Force scan across the lipid droplet for particle radius estimation (Methods). The lipid droplets vary in size from the trapping-force Rayleigh (dark red) and Mie (light red) limits. *N* = 2 droplets, representative for all the measurements. **d**, Fluorescence of *GFP*::*lmn-1* and bright-field images demonstrating nuclear deformation with a trapped lipid droplet on contact during a tweezer experiment (i). *δ* indicates the deformation of the nucleus during the test, and the arrowhead points to the trapped lipid droplet. Scale bar, 2 µm. Kymograph of two consecutive step indentations of an intestinal nucleus using a lipid droplet as the force probe (ii). Force and displacement during the same step of the indentation protocol (iii). **e**,**f**, Frequency-dependent shear modulus for two different ages of wild-type (**e**) and age-matched (**f**) *lem-2* mutants. The median and ±25% quantiles are represented by lines and shadows, respectively. **g**, Viscosity (*C*_*β*_) of the cytoplasm as extracted from the high-frequency component derived from the fit of the fractional Kelvin–Voigt model to the rheological spectrum of day 1 and day 8 adults for four different genotypes, as indicated. The red circle indicates median ± bootstrapped 95% confidence interval. *P* = 0.003 derived from a non-parametric Kruskal–Wallis test, followed by a pairwise comparison using a one-sided Dunn test without adjustment, as indicated above the horizontal brackets (for details on statistics and number of measurements, see Supplementary Data Table 2 and Extended Data Fig. 10). For wild type in D1, *N* = 35, *n* = 3, *m* = 10; in D8, *N* = 35, *n* = 3, *m* = 7. For *GFP*::*lmn-1* in D1, *N* = 32, *n* = 4, *m* = 9; in D8, *N* = 24, *n* = 4, *m* = 8. For *lem-2* in D1, *N* = 28, *n* = 3, *m* = 8; in D8, *N* = 20, *n* = 2, *m* = 6. For *emr-1* in D1, *N* = 25, *n* = 3, *m* = 10; in D8, *N* = 21, *n* = 2, *m* = 6. Source data

Aging is a multifactorial process under genetic control^40^, but how organismal age affects the mechanical properties of cells and tissues is not known. Thus, we applied TimSOM in specific tissues of *C. elegans*, and specifically asked if alterations of nuclear envelope proteins implicated in premature aging disorders^41,42^ influence the rheological properties of the cytoplasm during the first eight days of adulthood. Due to the difficulty in introducing microspheres into adult tissues without affecting animal physiology, we first established the use of endogenous lipid droplets as mechanical stress probes. Cellular lipid droplets resist deformation when subjected to forces^43^, and can itself cause nuclear indentation^43,44^ and even rupture the nucleus without being deformed^45^. Such droplets are abundant in various *C. elegans* tissues, including the intestinal epithelium (Fig. 5a) and the epidermis and, thus, have great potential as endogenous force probes.

To be suitable as optical tweezer probes, we confirmed that (1) their refractive index (*n*) is higher than the surrounding cytoplasm (Fig. 5b), (2) they are ~1 µm in diameter to maximize trapping stiffness^12,46^ (Fig. 5c) and (3) they are sufficiently stiff to be able to indent the desired target^43,45^. We, thus, isolated lipid droplets from *C. elegans* adults^47^ and used iodixanol as the index-matching media^48^ to determine their refractive index. At an iodixanol concentration of 48%, the droplets were indistinguishable from the surrounding medium, which corresponds to *n* = 1.42 (Fig. 5b). Thus, the lipid droplet’s *n* was larger than that of the cytoplasm (*n* = 1.33–1.37 (ref. ^49^)). We next used purified droplets as rheological probes in vitro and embedded them into PAA gels together with polystyrene microspheres as the control probes. We then compared the *G* modulus of the PAA gel obtained through TimSOM with both probes. The *G* modulus of PAA gels measured with lipid droplets coincided with that obtained from the embedded polystyrene microspheres (Extended Data Fig. 9). To confirm the suitability of lipid droplets as indenters in vivo, we verified that they can be trapped and moved in situ and can cause a visible deformation of the nuclear envelope when they are moved against the nucleus with up to 200 pN of contact force (Fig. 5d). Together, this indicates that endogenous lipid droplets are suitable as nanoindenters for optical force measurements.

We then trapped a lipid droplet and performed TimSOM in the cytoplasm of intestinal epithelial cells of young (day 1) and old (day 8) adults. The rheological spectrum was well modelled as a fractional Kelvin–Voigt material, indicating that the cytoplasm can be described as a viscoelastic solid. Generally, the cytoplasm was ten times stiffer than the cytoplasm of zebrafish progenitor cells (compare *C*_*α*_ and *C*_*β*_; Fig. 4 with Extended Data Fig. 10 and Fig. 5 with Extended Data Fig. 5), which prevented us to move the droplet within the cytoplasm to probe multiple compartments with the same trapped object. This solid-like signature was significantly reduced in old animals, suggesting that the viscoelastic properties of the cytoplasm fluidize during aging (Fig. 5e). In an attempt to define the genetic factors that contribute to cytoplasmic aging, we performed TimSOM in mutant strains causing envelopathies and premature aging-associated phenotypes^50,51^.

We specifically tested a dominant negative *GFP*::*lmn-1*/lamin A construct, and mutations in *emr-1* emerin and *lem-2* LEMD2 whose human homologues are implicated in muscle waste disorders and progeria^52^. These proteins localize to the inner nuclear membrane and are involved in coupling heterochromatin to the nuclear envelope with proposed roles in nuclear mechanics^53–55^. Their role in regulating the cytoplasmic rheology, however, is not understood. When we tested *GFP*:*lmn-1*/*lamin A*, we found generally lower storage and loss moduli over all the frequencies (Extended Data Fig. 10a) in young adults. In particular, the cytoplasmic viscosity of *lmn-1* animals, represented by parameter *C*_*β*_ (Fig. 5g), was significantly reduced to levels seen in older wild-type animals, that did not change further in the oldest specimens. Counterintuitively, we also observed a slight but insignificant increase in the elastic response in young animals of *lmn-1* visible in the reduction of the low-frequency exponent *α* compared with wild-type animals (Extended Data Fig. 10f). Together, this shows that defects in *lmn-1* cause the premature aging of the cytoplasmic rheology by a yet-unknown mechanism.

To further test the role of the nuclear envelope proteins in regulating cytoplasmic viscosity during aging, we tested *lem-2* and *emr-1* mutant animals. To our surprise, we did not observe major differences in cytoplasmic viscosity *C*_*β*_ compared with the control cells in young animals (Fig. 5e). Old animals had a slightly reduced viscosity, especially in animals lacking functional emerin EMR-1. In addition, during aging, emerin *emr-1* mutants exhibited a more fluid-like character, indicated by significantly higher exponents *α* and *β* (Extended Data Fig. 10f,g). Together, this suggests a general softening and fluidification of the cytoplasm during aging, which is accelerated by defects of the nuclear envelope (Extended Data Fig. 10 and Supplementary Text 3).

## Conclusions

Here we introduced an active microrheology procedure based on optical gradient traps, optimized for small sample volumes, which massively increases experimental throughput by reducing material expenditure, instrumentation complexity and measurement time.

Our method has important advantages over existing active rheology routines based on two separate driving and detection lasers^21,56^, or separating one laser beam into two beams using orthogonal polarization^57^. The use of a single laser for force and position measurements reduces the complexity in aligning two optical paths and two detectors at the BFP interferometry collection system and ensuring constant ‘alignment’ and thus rheological experiments over the whole operational field of the trap.

Our examples highlighted that TimSOM correctly measured the previously described Maxwell-like behaviour of BMCs and their time-dependent rigidity transition, but also to decipher the intracellular mechanics of different organelles and dramatic alterations of cytoplasmic properties in mutants known to accelerate the animal aging process.

Despite the demonstrated versatility, our approach has some shortcomings that arise from the violation of the time continuity intrinsic in an AOD-based time-sharing configuration. First, the response of the probe at high frequencies is underestimated if the elastic contribution of the material is too large. Thus, this deviation depends on the type of material that is being probed. We developed a detailed protocol and a compensation algorithm to correct for this deviation, which requires a basic assumption on the underlying material properties of the sample, for example, if it behaves primarily like a viscoelastic liquid or viscoelastic solid. Importantly, as shown here (Figs. 4 and 5) and by other work^1,19–21,58^, cells primarily behave as a fractional Kelvin–Voigt material, which we found to be less affected by this deviation. For ideal Maxwell liquids in an arbitrarily stiff trap, the time-sharing method cannot unequivocally determine the real part of the response function in the frequency spectrum. Thus, it may be necessary to make the measurements with a trap that is less rigid than the material behaviour at the Nyquist frequency (Supplementary Text 1.10).

Together, our examples illustrate how TimSOM can be used to measure various materials, ranging from simple viscous fluids to the complex response of viscoelastic fluid-like protein condensates and different organelles inside living cells and whole animals.

## Methods

All research complies with the relevant ethical regulations. For experiments on zebrafish, all protocols used have been approved by the Institutional Animal Care and Use Ethic Committee (PRBB–IACUEC) and implemented according to national and European regulations. *C. elegans* is a nematode and, thus, exempt from ethical approval and regulations.

### Theory and simulation of the bead displacement in a viscoelastic medium in a time-varying trapping potential

The effect of having two alternating optical traps on the actual measurement of the response function *χ*_t_(*ω*), and, therefore, *G* modulus *G*(*ω*), is introduced in this section and detailed in the Supplementary Information. Two methods are introduced. First, we use a numerical method in the time domain, aiming at the trajectory of the trapped probe, to emulate the violation of the continuity condition between the force *F*(*t*) = *F*_1_(*t*) + *F*_2_(*t*) and position *x*_p_ = *F*_2_/*k* measurements (Fig. 1). Second, by truncating the dynamic, oscillatory trapping potential at the first harmonic, we obtain an analytical expression in the frequency domain that we further use to compensate the time-sharing effect, that is, to retrieve *χ*(*ω*) from *χ*_t_(*ω*).

#### Study of deviation via FDEs

Under an AOD-modulated time-sharing regime, the laser spot gradually vanishes from trap 1 and appears into trap 2 as the acoustic wave enters the laser beam cross-section at the AOD crystal (Supplementary Information). In our setup, this occurs within a transition time of *τ* = 10 µs. After that, the laser spot remains at trap 2 for 30 µs and the transition is, therefore, reversed back into trap 1. Meanwhile, a voltage data point is sampled with a delay of 33 µs with respect to the rising edge of the acoustic wave. Because traps 1 and 2 fall within the linear regime of the trapped particle, forces acting onto it are equivalent to that from a single trap undergoing the following trapezoidal trajectory (Supplementary Information):

2 $${x}_{l,n}(t)=\left\{\begin{array}{ll}\begin{array}{ll}{x}_{2,n}-\frac{t}{\tau }\left({x}_{2,n}-{x}_{1,n}\right) & 0\le t < 10\,\upmu {\rm{s}}\\ {x}_{1,n} & 10\,\upmu {\rm{s}}\le t < 40\,\upmu {\rm{s}}\\ {x}_{1,n}-\frac{t}{\tau }\left({x}_{1,n}-{x}_{2,n}\right) & 40\,\upmu {\rm{s}}\le t < 50\,\upmu {\rm{s}}\\ {x}_{2,n} & 50\,\upmu {\rm{s}}\le t < 80\,\upmu {\rm{s}}\end{array} & n=0,\,1,\,2\ldots \end{array}\right.,$$

where *x*_2,*n*_ = 0, ∀*n* and *x*_1,*n*_ are discrete values of *x*_1_(*t*) = 2*x*_0_sin(*ωt*) sampled at *f*_t_ = 12.5 kHz. Here the amplitude of oscillation in *x*_1_(*t*) is expressed as *A* = 2*x*_0_ for mathematical convenience. To capture the power-law rheological behaviour of most samples in biology, we use the fractional time-derivative operator ${D}_{{\rm{t}}}^{\alpha }$, which describes the stress–strain relationship of a springpot as $\sigma (t)=C_{\alpha} {D}_{{\rm{t}}}^{\,\alpha }\epsilon (t)$ (ref. ^19^). We numerically solved equations (3)–(5) to obtain the trajectory of the probe bead in the laboratory frame *x*(*t*) for different materials and at different driving frequencies *ω*_*j*_. Therefore, the instantaneous force is obtained as *F*(*t*) = *k*(*x*_l_(*t*) – *x*(*t*)), from which the interleaved BFP interferometry voltage signals *V*_1_(*t*_1,*i*_) and *V*_2_(*t*_2,*i*_) are sampled at time points *t*_1,*i*_ and *t*_2,*i*_, respectively. Finally, the fast Fourier transform of the two signals is taken and the response function $\hat{\chi}_t(\omega_j)$ and *G* modulus $\hat{G}_t(\omega_j)$ are obtained using equations (1a) and (1b). A Python code (v. 3.9.7) is available to carry out the FDE simulations (Supplementary Information, https://gitlab.icfo.net/rheo/Tweezers/timsom). The comparison between the physical response function $\hat{\chi}(\omega_j)$ and the one accounting for the time-sharing deviation $\hat{\chi}_t(\omega_j)$ is shown in Fig. 1g for three different power-law materials. The corresponding *G* moduli (both physical and time-sharing deviated) are shown in Extended Data Fig. 1.345

### FHA

To correct the observed deviations in the FDE and the experiment, we analytically solved the equations of motion of the bead $\hat{x}(\omega')$ in the space of frequencies *ω*′:

6 $$\hat{x}\left({\omega }^{{\prime} }\right)=\hat{\chi }\left({\omega }^{{\prime} }\right)k\left[{\hat{x}}_{l}\left({\omega }^{{\prime} }\right)-\hat{x}\left({\omega }^{{\prime} }\right)\right],$$

which becomes

7 $$\hat{x}\left({\omega }^{{\prime} }\right)={\hat{\chi }}_{{\rm{a}}}\left({\omega }^{{\prime} }\right)k{\hat{x}}_{l}\left({\omega }^{{\prime} }\right),$$

Here $\hat{\chi}_a(\omega')$ is the the response function of the active–passive system (Supplementary Equation (3)). $\hat{x}_l(\omega')$ is the Fourier transform of the FHA of the trap trajectory, which—in the time domain *t*—reads as

8 $${x}_{{\rm{l}}}(t)=\frac{{x}_{1}}{2}\exp ({\rm{i}}\omega t)\left[1+\sin \left({\omega }_{{\rm{t}}}\cdot t\right)\right],$$

where the complex oscillation exp(i*ωt*) is used, instead of a real one such as sin(*ωt*) or cos(*ωt*), with the sole purpose of simplifying the notation in the frequency space. After Fourier transform, the trap trajectory reads

9 $${\hat{x}}_{{\rm{l}}}\left({\omega }^{{\prime} }\right)=\frac{{x}_{1}}{2}\left[\delta \left({\omega }^{{\prime} }-\omega \right)+\frac{\delta \left({\omega }^{{\prime} }-{\omega }_{+}\right)-\delta \left({\omega }^{{\prime} }+{\omega }_{-}\right)}{2{\rm{i}}}\right],$$

where *ω*_±_ = *ω*_t_ ± *ω* and *δ*(*ω*′) is the Dirac distribution.

To obtain the linear response function *χ*_t_(*ω*) from the FHA approximation, the same steps identified for the FDE simulation must be followed, but this time using an analytical approach. The details of the calculations are given in Supplementary Section 1.8. The obtained expression of $\hat{\chi}_t(\omega)$ is a function of the response function of the material $\hat{\chi}$, time-sharing frequency *ω*_t_ and trap stiffness *k*. It reads as

10 $${\hat{\chi }}_{{\rm{t}}}(\omega )=g\left(\hat{\chi },{\omega }_{{\rm{t}}},k\right)(\omega )=\frac{\hat{\chi }(\omega )-{\hat{\chi }}_{1}(\omega )+k\left[\;\hat{\chi }(\omega ){\hat{\chi }}_{1}(\omega )-{\hat{\chi }}_{+}(\omega ){\hat{\chi }}_{-}^{* }(\omega )\right]}{1+2k{\hat{\chi }}_{1}(\omega )+{k}^{2}{\hat{\chi }}_{+}(\omega ){\hat{\chi }}_{-}^{* }(\omega )},$$

where

11a $${\hat{\chi }}_{1}(\omega )=\frac{1}{2}\left[{\hat{\chi }}_{+}\left(\omega \right)+{\hat{\chi }}_{-}^{\,* }\left(\omega \right)\right],$$

11b $${\hat{\chi }}_{+}(\omega )=\hat{\chi }\left({\omega }_{{\rm{t}}}+\omega \right),$$

11c $${\hat{\chi }}_{-}^{* }(\omega )={\hat{\chi }}^{* }\left({\omega }_{{\rm{t}}}-\omega \right)=\hat{\chi }\left(-{\omega }_{{\rm{t}}}+\omega \right).$$

The asterisk ‘*’ denotes the complex conjugate operation.

The goodness of the FHA approximation was then checked by comparing the results predicted using equation (10) with those predicted using the FDE approach. To do so, we applied the FHA correction to the deviated data simulated through FDE (Fig. 1g).

### Optical micromanipulation and fluorescence microscopy

#### Optical tweezers

Our optical micromanipulation and microscopy platform is built around an inverted microscope (Nikon Ti2) with a spinning-disc module (Andor DragonFly 505)^23^. The optical tweezer unit (SENSOCELL, Impetux Optics) is coupled to the rear epifluorescence port of the Ti2 microscope. The 1,064 nm (maximum output power, 5 W) trapping laser is modulated by a pair of AODs (for *x*- and *y*-axes control) optically conjugated to the entrance pupil of a water-immersion objective (Nikon Plan Apo, ×60, numerical aperture = 1.2), which, in turn, focuses the trapping beam onto the focal plane, thereby generating the optical traps. The AODs are addressed at a frequency of 25 kHz, for which each trap is addressed at 12.5 kHz in the dual-trap time-sharing configuration and hence the maximum oscillatory frequency for active microrheology results is 6.25 kHz (Nyquist frequency).

#### Force and position detection with time-sharing optical tweezers

Optical force measurements were performed with a BFP interferometry system (SENSOCELL, Impetux Optics), optimized for light momentum detection^59^. The forward scattered light is captured using a high-numerical-aperture condensor and conveyed to a position-sensitive detector for which volt-to-piconewton conversion factor *α* is calibrated by the manufacturer. Probe positions—relative to the trap—were derived after measurements of the trapping stiffness *k* (pN µm^–1^) by fast scanning the trap across the probe. Variations in the initial light momentum and trap power over the field of view are compensated through the driving software of the optical traps (LightAce v. 1.6.2.0 SDK for acquiring optical tweezer force spectroscopy and for active microrheology, Impetux Optics). A detailed protocol for the startup and use of this optical tweezer platform can be found elsewhere^23^.

#### Active microrheology

Our active microrheology measurements consist of four steps.

- Centering the laser on the trapping probe using the ‘Particle Scan’ routine of the LightAce software: this is only needed for solid samples. Conveniently, for primarily liquid samples (water or protein droplets), the bead is pulled into the trapping potential without any centring routine.
- Choice of *G*_0_ (Supplementary Text 7.1)
- Measure the trap stiffness: to do this, the laser is scanned across the trapping probe. A linear fit to the linear regime, within −200 nm ≤ 0 ≥ 200 nm, was applied. The slope of the line directly provides *k* through a link between force (*F*, as measured through momentum changes on the position-sensitive detector^22^) and trap position *x*_*l*_ such that *k* = *F*/*x*_*l*_.
- Apply a series of oscillations with specific parameters of frequency, amplitude and measurement duration. The frequencies and amplitudes applied in every measurement are specified in Supplementary Tables 3–5
- Retrieval of χ(ω) and *G*(*ω*) values from $\hat{\chi}_t(\omega_j)$ through the FHA method (RheoAnalysis, Impetux Optics).

### Microrheology in calibrated materials

#### Glycerol solutions

Different concentrations of glycerol (from 0% (= MilliQ water) to 95%) mixed with a fluorescent bead solution (1 µm microspheres, Thermo Fisher, F8816) were used. Parameters for the rheology routine are provided in Supplementary Table 3. The viscosity *η*_TimSOM_ for the glycerol mixtures was obtained from the slope of the loss modulus *G*″(*ω*) = *η*_TimSOM_*ω* (Fig. 2a and Extended Data Fig. 2a(iii)). Stokes-drag force measurements were performed by moving the same bead with increasing velocities, *v*_trap_ (Extended Data Fig. 2a). The constant plateau force during the bead movement, *F*_drag_, was related to the velocity. The viscosity *η*_drag_ was determined by fitting *F*_drag_(*v*_trap_) = (6π*η*_drag_ *Rv*_trap_) to the force–velocity plots, where *R* is the radius of the trapped microsphere (Extended Data Fig. 2a(iii)). To avoid changes in viscosity due to laser absorption, trapping power was left below *P* = 65 mW (ref. ^60^). To avoid hydrodynamic interaction with the chamber surfaces, the trapping plane was kept at a relative height of *z* = 20 µm (ref. ^61^).

#### PAA gels

PAA gels were polymerized from a modification of the recipe used in ref. ^62^. Exact quantities for obtaining specific gel stiffness are tabulated in Supplementary Table 4 and the protocol to produce the gel is detailed in Supplementary Text 6. Before the microrheology measurement, the probe/trap centring routine, followed by the trap stiffness measurements, was carried out. The fractional Kelvin–Voigt model was fit to the obtained rheological spectrum (Fig. 2c) to extract the relevant parameters and compared with existing literature^63^.

#### Creep compliance measurements

Creep compliance measurements were performed on 2% PAA gel using force-clamp utility of the LightAce software (LightAce v. 1.6.2.0 SDK, Impetux, Spain). We used a trap stiffness of 890 pN µm^–1^ and 220 pN µm^–1^ for stiff and soft gels, respectively. A constant force of *F*_0_ = 40 pN was applied, which leads to a typical viscoelastic compliance curve *x*(*t*) (Extended Data Fig. 2b). A custom MATLAB code was implemented to calculate the frequency-dependent shear modulus from measurements of *J*(*t*) in the time domain^25^. The position of the trapped probe was calculated as *x*_bead_ = *x*_trap_ − *F*/*k*. The low-frequency plateau modulus was calculated from the frequency-dependent storage *G* modulus after fitting to a fractional Kelvin–Voigt model (Fig. 2c(ii)).

#### PDMS

PDMS prepolymer was mixed with a curing agent at 100:1 and mixed thoroughly with *d* = 1 µm microspheres (F8816, Thermo Fisher). The solution was degassed in a vacuum pump to eliminate bubbles introduced during mixing. The mixture was immediately used for rheology to avoid the long-term curation of silicone. Before rheology measurements, the probe/trap centring routine was performed using the LightAce software. Further details are available in Supplementary Text 6.

### Zebrafish experiments

#### Microsphere injection and cell preparation

Zebrafish embryos were injected with 1 nl of microspheres (F8816, Thermo Fisher; diameter, *d* = 1 µm) at 1:5 of the stock solution at the one-cell zygote stage to ensure proper distribution of microspheres^23^. Microspheres were coinjected with mRNAs (Supplementary Text 6). At 4 h post-fertilization, embryos were dechorionated with a pair of forceps and their cells were manually dissociated and left to recover until used^64^. To visualize the nucleus, the dissociated cells were incubated for 6 min in DNA Hoechst at a final concentration of 1 µg ml^–1^ following the protocol in another work^23^. A layer of double Scotch tape (approximately 20 × 20 mm^2^ wide) was used as a spacer between the lower and upper surfaces. A 1 × 1 cm^2^ hole was made and the layer was adhered onto the bottom dish (GWST-5040, WillCo). After incubation with concanavalin A to promote cell adhesion (*t* = 30 min, 100 µl, 0.5 mg ml^–1^; C5275 Sigma), cells were allowed to settle onto the bottom dish surface^65^ and the cavity was covered at the top with a 22 × 22 cm^2^ cover glass (Ted Pella).

#### Active microrheology measurements in zebrafish progenitor stem cells

To perform active microrheology measurements inside a cell, first, a cell with one or two microspheres in the cytoplasm is selected. Then, a working optical plane is identified looking at nucleus fluorescence, where the nucleus has its biggest cross-section. Subsequently, the microsphere is trapped and placed in the selected plane between the plasma and nuclear membrane, avoiding adhesions to either one. After placement, the active microrheology is initialized (Supplementary Table 5 lists the parameters).

After the measurement of the cytoplasm is completed, the bead is placed to be in contact with the nuclear envelope. This is done by slowly moving the microsphere towards the nucleus and observing the force signal due to bead displacement (Supplementary Video 3). A force peak is visible once the bead touches the nucleus. Then, the microrheology routine was performed perpendicular to the nuclear envelope surface. All active microrheology measurements are performed using a trap power of 60–100 mW at the sample plane and were found to not influence the sample behaviour (Supplementary Text 5.1).

#### Microsphere insertion into the nucleus

The process of inserting the microsphere inside the cell nucleus consists of applying a constant force using the optical force feedback system explained above (force setpoint, *F* = 100−200 pN, Extended Data Fig. 7). In this way, the microsphere is pushed into the nucleus. First, the bead is placed in contact with the nucleus at the optical plane in which its fluorescent cross-section is the biggest. Then, the clamp is set from 100 pN to 150 pN and it is activated until the bead is inserted, or until the microsphere does not indent more or gets lost. The first 5 s of the bead trajectory, and before insertion, were used to calculate the creep compliance of the nucleus. Finally, after the bead was inserted in the nucleus, a rheology routine was performed inside the nucleus. The oscillations of this measurement were done perpendicular to the insertion direction.

##### Analysis of creep compliance data

Microsphere displacement under clamping force was analysed as a creep compliance routine fitted with a Jeffrey’s model using a custom Python script (v. 3.10). All the experimental routines were analysed, including those where insertion was unsuccessful. For successful insertion trials, only the initial phase—during which the bead indents but does not penetrate the nucleus—was considered. For these routines, the normalized position was fitted using^66^

12 $$\frac{x\,(t)}{{f}_{{{\rm{FC}}}}}=\frac{1}{\kappa }\left(1-{{\rm{e}}}^{-\frac{\kappa }{{\gamma }_{1}}t}\right)+\frac{t}{{\gamma }_{2}},$$

where *x*(*t*) is the displacement of the microsphere, which is divided by clamping force *f*_FC_. This normalization compensates for variations in the forces used for bead insertion in different cells. The fittings allow the extraction of restoring stiffness *κ* and viscoelastic drags *γ*_1_ and *γ*_2_.

### Experiments in *C. elegans*

#### *C. elegans* maintenance

Strains were maintained and manipulated under standard conditions^67^. Nematode strains were grown at 20 °C on nematode growth medium plates with OP50 bacteria and synchronized using the standard alkaline hypochlorite treatment method^68^. Only age-matched, synchronized adult hermaphrodites (day 1 or day 8) were used in this study. N2 wild-type strain was used as a control, unless otherwise stated.

#### Lipid droplet isolation

*C. elegans* lipid droplet isolation was performed following the protocol previously described^47^ with minor modifications. Briefly, for sample preparation, MSB1136 animals were grown on peptone-enriched plates seeded with NA22 bacteria and synchronized using the standard alkaline hypochlorite treatment method^68^. Then, 2 × 10^4^ synchronized larva-1-stage nematodes were plated and grown at 20 °C until either day 1 or day 8 adulthood for sample collection. For lipid droplets, day 8 collection, animals were washed with M9 and separated from laid eggs by gravity (to avoid generation mixing) before transferring them to new plates. On the day of the experiment, plates were washed off, and animals were collected using phosphate-buffered saline. After washing the sample three times with buffer A^47^, the pellet was resuspended in the same buffer supplemented with a protease inhibitor (Sigma-Aldrich, P8340). After this, the sample was manipulated either on ice or at 4 °C. Nematode homogenization and cell disruption were performed by using an ultrasonic bath (VWR, USC300TH) four times, 1 min each time, with 30 s intervals. Lipid droplets were collected in buffer B^47^ from the post-nuclear fraction by ultracentrifugation followed by three washing steps. Isolated lipid droplets were used within the same day for active microrheology routine in PAA gels (see the section above).

#### Refractive-index-matching assay

The refractive index of freshly isolated lipid droplets was measured as described^48,69^ using the commercially available iodixanol solution (OptiPrep, D1556, Sigma-Aldrich). Lipid droplet solution was gently mixed at different iodixanol concentrations until reaching the matching refractive concentration (48%) and 0.5% membrane dye BioTracker NIR750 (Sigma-Aldrich, SCT113). For the optical trap scanning and imaging of the lipid droplets, the sample was mounted in an optical trapping chamber and then sealed with a #1.5 cover glass, as previously described^23^.

#### Lipid droplet characterization by TimSOM in vitro

To prepare PAA gels of different stiffness values containing both lipid droplets and microspheres, freshly isolated lipid droplet solution was gently mixed with the reagents noted in Supplementary Table 4. The water was substituted by the same volume of lipid droplet solution for each class of PAA gel. The mix was transferred into the optical trapping cavity and then sealed with a #1.5 cover glass, as previously described^23^. To extract both lipid droplet size and stiffness values, the measurements were performed alternatively using lipid droplets and microspheres following the procedure detailed above.

#### In vivo TimSOM on *C. elegans* intestinal epithelial cells during aging

All strains were seeded on nematode growth medium plates on the same day and allowed to grow until day 1 (three days post-seeding) and day 8 (ten days post-seeding) adulthood. The animals evaluated on day 8 were transferred to new plates every day during the egg-laying period to avoid mixing generations. Because both LW697 and BN20 presented one-day developmental delay compared with wild type and BN19, active microrheology measurements on day 1 and day 8 adulthood for those strains were performed 4 days and 11 days post-seeding, respectively. On the day of the experiment, nematodes were mounted on 2% agar pads, immobilized with 10 µM levamisole hydrochloride solution (Sigma-Aldrich, 31742) and then covered with a 25 × 25 mm^2^ cover glass (Ted Pella, #1.5) sealed with fingernail polish. Active microrheology was performed, as described above, for zebrafish progenitor stem cells on *C. elegans* intestinal cells using endogenous lipid droplets within 1 h after immobilization to avoid damage to the animals.

### Data analysis

Analysis of the data measured using active microrheology is performed in RheoAnalysis (Impetux Optics). In this program, the complex shear modulus is retrieved by performing the required compensations (Supplementary Text 1.12). Furthermore, fittings from the first two third-order models can be applied to the dataset by selecting the respective initial conditions. The analysis of other calibration experiments such as the Strokes-drag force measurements, creep compliance measurements and simulations was done on personalized MATLAB (v. 2020b) and Python (v. 3.10) scripts.

### Statistics and reproducibility

Statistical modelling and hypothesis testing were performed in MATLAB (v. 2020b) and R (v. 4.2.2). No statistical methods were used to predetermine the sample sizes, but our sample sizes are similar to those reported in previous publications. Data distributions were assumed to be normal, unless obviously not, but this was not formally tested. All the paired measurements were tested with a paired, two-sided *t*-test. All datasets were acquired in a randomized fashion, and when they were not (for example, frequency sweep in the rheology routine), the data were not biased by the history. Data were not collected blind to the genotype, but the data presented in Fig. 5 were analysed blind to the experimental condition. Negative data points in the rheological spectrum were excluded from the fitting procedures.

### Reporting summary

Further information on research design is available in the Nature Portfolio Reporting Summary linked to this article.

## Online content

Any methods, additional references, Nature Portfolio reporting summaries, source data, extended data, supplementary information, acknowledgements, peer review information; details of author contributions and competing interests; and statements of data and code availability are available at 10.1038/s41565-024-01830-y.

## Supplementary information

Supplementary Information Supplementary Texts 1–12, Figs. 1–33, Equations (1)–(91), Tables 1–7 and references.

Reporting Summary

Supplementary Tables 1–7 All tables in one file, each on a different sheet/tab.

Supplementary Video 1 Representative video of the dual-trap assay to measure the surface tension and mechanical properties of the protein droplet. Scale bar, 5 µm.

Supplementary Video 2 Representative video of the TimSOM assay to measure the bulk mechanical properties of the protein droplet. Scale bar, 5 µm.

Supplementary Video 3 Representative video of the complete rheology routine inside a zebrafish progenitor cell. Scale bar, 10 µm.

Supplementary Video 4 Representative video of a zebrafish progenitor cell subjected to a bead insertion routine, after applying a 100 pN force clamp for ~5 s. Force clamp started after 5 s and entered the nucleus after 11 s, after which the force-clamp routine is abruptly stopped. Scale bar, 10 µm. Green, lap2beta; blue, Hoechst 33342. Time label is shown in seconds.

Supplementary Video 5 Representative video of a zebrafish progenitor cell stained with Hoechst 33342 to highlight the nuclear DNA, which expresses nuclear-localized, soluble GFP. No obvious leakages of DNA and soluble GFP from the nucleus into the cytosol after bead insertion are visible, indicating no substantial damage to the nuclear envelope. Video representative for *N* = 3 cells. Scale bar, 10 µm. Blue, Hoechst 33342; green, nuclear-localized, soluble GFP.

Supplementary Video 6 Representative video of a dividing zebrafish progenitor cell. Scale bar, 10 µm.

Supplementary Video 7 Representative video of a dividing zebrafish progenitor cell after the insertion of a microsphere into the nucleus. Scale bar, 10 µm.

## Source data

Source Data Fig. 1 Statistical source data.

Source Data Fig. 2 Statistical source data.

Source Data Fig. 3 Statistical source data.

Source Data Fig. 4 Statistical source data.

Source Data Fig. 5 Statistical source data.

**Extended Data Fig. 1:**
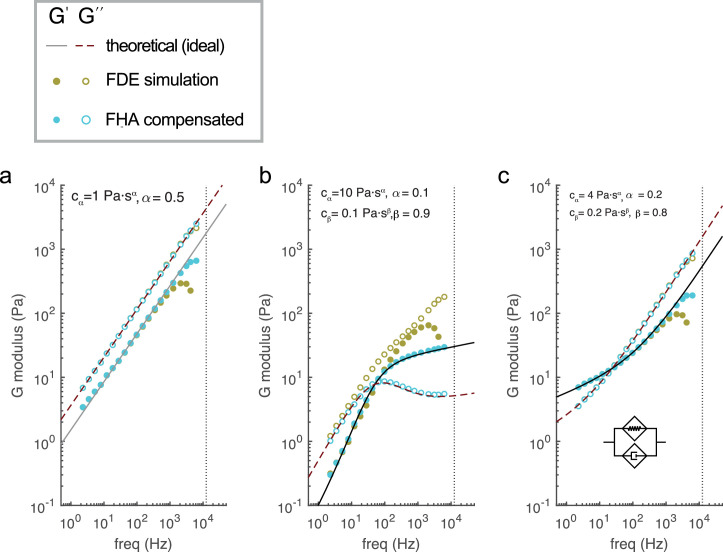
Theoretical analysis of the compensation of the G modulus extracted from TimSOM. **a-c**, G moduli calculated from the response function (Eq. 1), ${G}^{*}=\frac{1}{6\pi R{\chi }^{*}\left(\omega \right)}$ simulated through the FDE method for the (**a**) single springpot model, (**b**) the fractional Maxwell and the (**c**) fractional Kelvin-Voigt model. Parameters shown in the panels were used to during the FDE simulation. Source data

Source Data Extended Data Fig. 1 Statistical source data.

**Extended Data Fig. 2:**
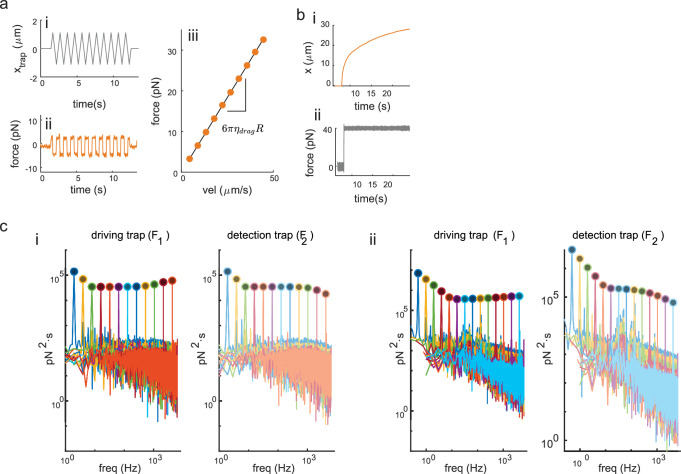
TimSOM accurately measures viscous and viscoelastic properties. **a**, Example of a triangular trajectory (i) used to generate constant drag forces (ii) to measure the viscosity of a purely viscous material. Viscosity was obtained from a linear fit (solid line, iii) to the averaged force values obtained for a series of triangles with different velocities (orange dots, iii). The viscosities derived from different methods for increasing glycerol concentrations are plotted in Fig. 2. **b**, Force clamp experiment to retrieve the G modulus of a soft PAA gel. The creep motion of the trapped bead (*J*(*t*), i) upon a feedback-controlled constant force (*F*_0_ = 40 pN, ii) can be used to retrieve the frequency- dependent G modulus shown in Fig. 2. **c**, Fourier and frequency domain analysis of TimSOM force signals. Power spectrum obtained from the force signals of the driving (*F*_1_) and static traps (*F*_2_) during an active microrheology measurement on a 1-µm microsphere in water (i, *f* = 2*,…*, 4096 Hz) and in a 21.1 Pa PAA gel (ii *f* = 0.5*,…*, 4096 Hz). The peaks are located at the imposed driving frequencies. The signal is clearly distinguishable and larger than the background noise. Source data

Source Data Extended Data Fig. 2 Statistical source data.

**Extended Data Fig. 3:**
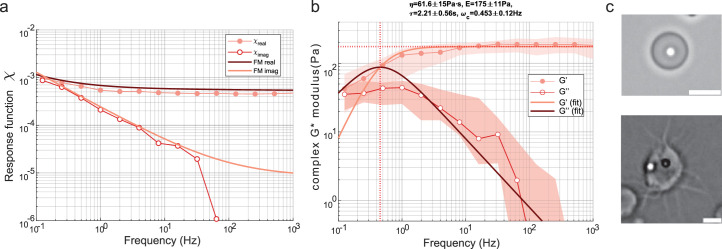
Rheology of CPEB4 droplets. **a**, Response function acquired on naive CPEB4 condensates immediately after formation. Thick lines correspond to the fit of the data to a fractional Maxwell model **b**, Rheological spectrum of CPEB4. Solid lines indicate the fit to a Maxwell model. G’, storage modulus; G”, loss modulus. Fit parameters are indicated on top of the graph: *η*, viscosity; E, plateau modulus; *τ*, relaxation time; *ω*_*c*_, crossover frequency. Mean ± standard deviation of N = 9 droplets (for each timepoint) acquired on three independent experiments. **c**, Image (representative for N = 5 droplets) of a fresh (top) and an ‘aged’ CPEB4 condensate (bottom) displaying emerging fibres indicating their solid transition. Scale bar = 5 µm. Source data

Source Data Extended Data Fig. 3 Statistical source data.

**Extended Data Fig. 4:**
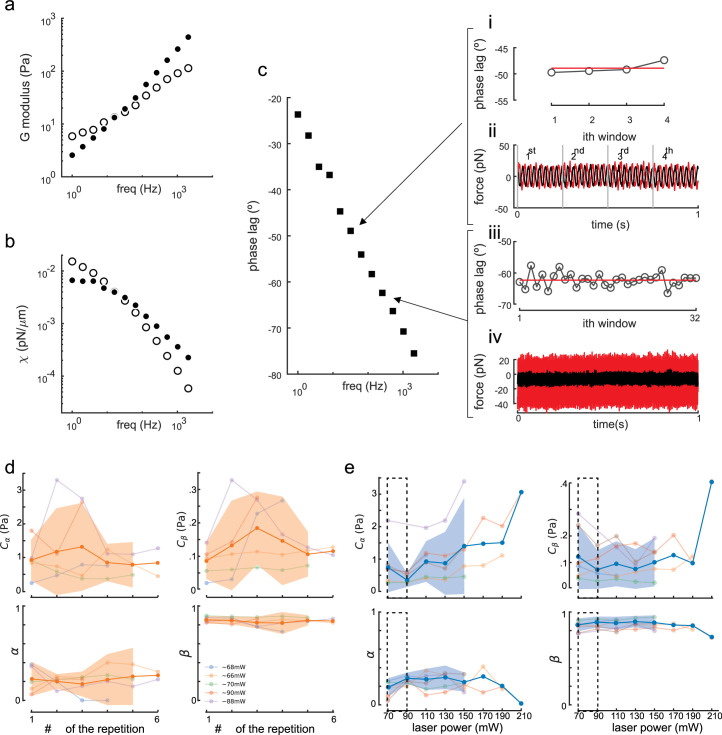
Performing the rheology routine does not induce phase lag difference in zebrafish cells. **a, b**, Representative measurement of the G modulus (a) and response function (b) on a microsphere in the cytoplasm of a zebrafish embryonic cell. **c**, Performing the rheology routine does not induce phase lag difference. Phase lag between the position and the force oscillatory signals. In inset i, the phase lag over consecutive time windows containing 8 cycles (inset ii) is shown for the oscillation at 32 Hz. In insets iii and iv, same as in i and ii, for the oscillation at 256 Hz. **d**, Performing rheological routines repeatedly in the same cell with the same power does not elicit a noticeable history effect. Each curve is acquired at the indicated laser power in the same cell. Solid curve = mean; shaded area is 95% confidence interval. **e**, Performing rheological routines repeatedly with increasing laser power induces substantial stiffening in the cell, noticeable through an increase in *C*_*α*_. N = 6 cells. Solid curve = mean; shaded area is 95% confidence interval. The value of 90 mW in cell 1 was omitted as the fit to the fKV model did not converge. For clarity and convenience, we choose to start with 70 mW which provided enough trapping strength to resist cytoplasmic motion caused by ‘circus bleb movements’ in these cells. The dotted box indicates the powers at which the measurements have been done throughout this manuscript. Source data

Source Data Extended Data Fig. 4 Statistical source data.

**Extended Data Fig. 5:**
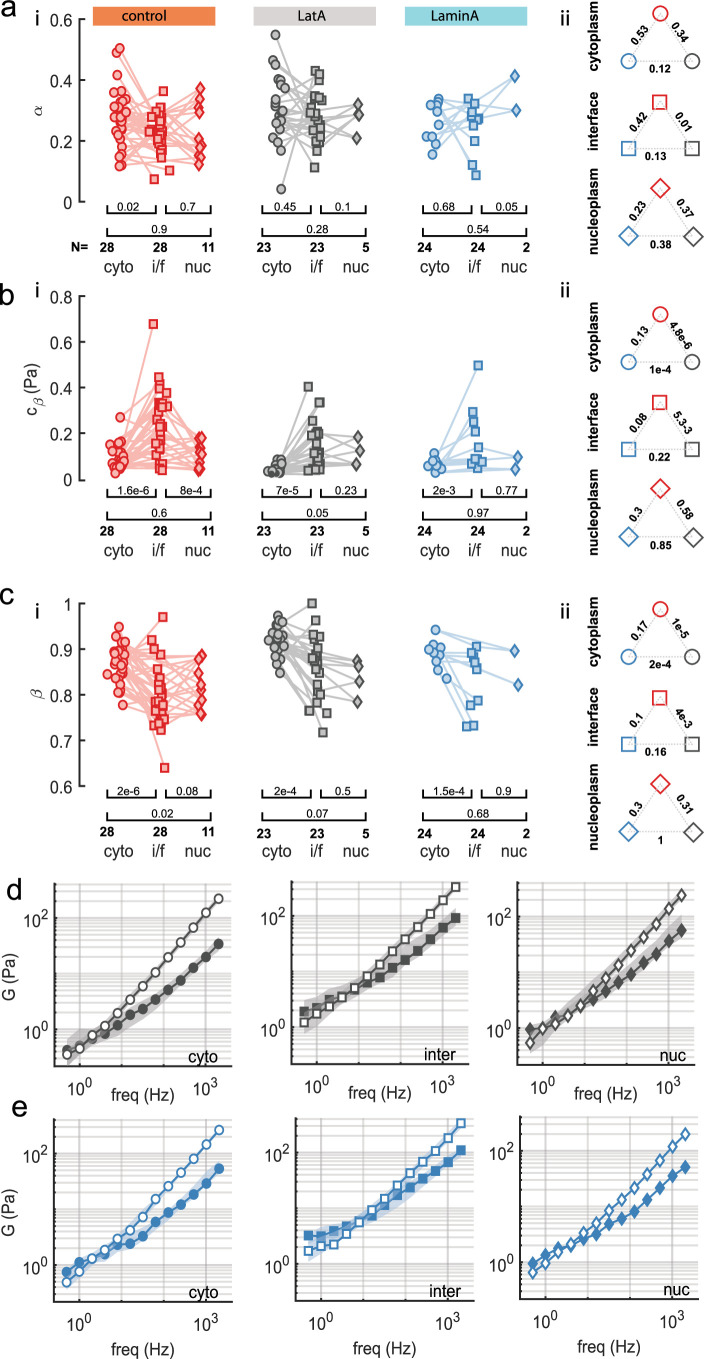
Active microrheology of zebrafish progenitor cells. **a**, (i) Exponent *α* of the low-frequency component derived from the fit of the fractional Kelvin-Voigt to the rheological spectrum in Fig. 4; (ii) p-values derived from a pairwise, two-sided Mann-Whitney U-test of the indicated combinations. **b**, (i) Prefactor *C*_*β*_ of the high-frequency component derived from the fit of the fractional Kelvin-Voigt to the rheological spectrum in Fig. 4. (ii) p-values derived from a pairwise, two-sided Mann-Whitney U-test of the indicated combinations. **c**, (i) Exponent *β* of the high-frequency component derived from the fit of the fractional Kelvin-Voigt to the rheological spectrum in Fig. 4. (ii) p-values derived from a pairwise, two-sided Mann-Whitney U-test of the indicated combinations. p-values below the graph in panels (i) derived from a two-sided, paired t-test; N indicates the number of cells measured. **d, e**, Rheological spectrum acquired on the cytoplas, nuclear interface and the nucleoplasm derived from (d) latrunculin A treated cells and (e) Lamin A overexpressing cells. Source data

Source Data Extended Data Fig. 5 Statistical source data.

**Extended Data Fig. 6:**
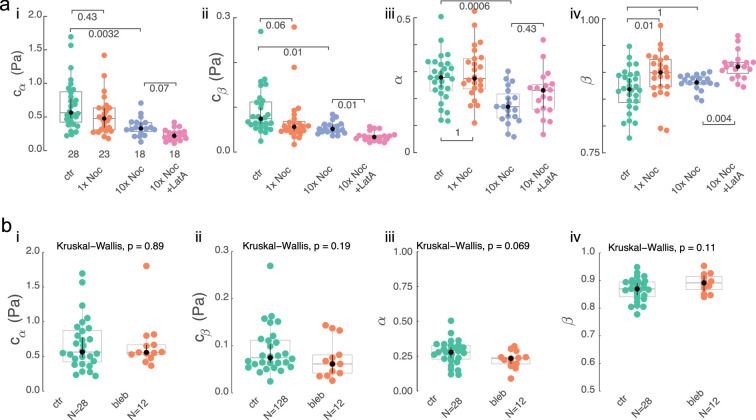
Cytoplasmic rheology is affected by tubulin organization but unaffected by myosin II activity. **a, b**, Dot plot of all four parameters extracted from a fit of the rheological spectrum to a fractional Kelvin-Voigt model comparing the cytoplasm of untreated control cells with cell treated with varying concentrations of Nocodazole (1 µM and 10 µM) to depolymerize the microtubule cytoskeleton and combined LatA/Nocodazole to interfere with actin and microtubule network; and (b) 10 µM blebbistatin to inhibit myosin II contractility. (i) Prefactor *C*_*α*_ indicating magnitude of the low-frequency, elastic response; (ii) Prefactor *C*_*β*_ indicating magnitude of the high-frequency, viscous response; (iii) Exponent *α* indicating the low-frequency, solid behavior; (iv)) Exponent *β* indicating the high-frequency, fluid behavior. Number of independent experiments are n=9 (ctrl), 4 (1xNoc), 3 (10xNoc), 2 (10xNoc+LatA), 2 (bleb). Values close to the horizontal bracket indicate the p-value of (a) two-sided Kruskal-Wallis test with a Dunn post-hoc test for pairwise comparisons between groups with Bonferroni adjustment, and (b) unpaired, two-sided Kruskal Wallis test, comparing the two groups. In all panels, boxes indicates the central 50% of the data around the median (horizontal line), and the whisker delimit the 10^th^ and 90^th^ percentile. Source data

Source Data Extended Data Fig. 6 Statistical source data.

**Extended Data Fig. 7:**
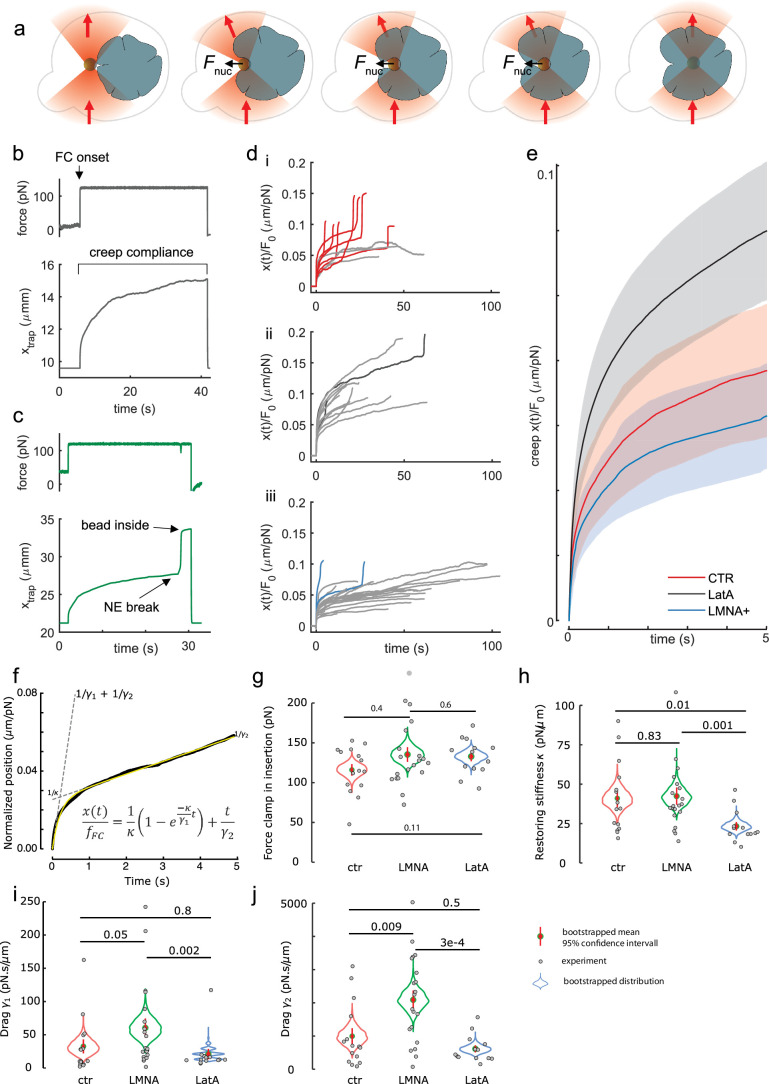
Force clamp protocol for microsphere insertion into zebrafish embryonic cell nuclei. **a**, Schematics of the nuclear insertion process using the force clamp modality of the optical micromanipulation platform. A bead is brought into contact with the nucleus and a force clamp is set at *F*_0_ = 100 ~ 150 pN. During some tens of seconds, the bead creeps against the nuclear envelope, eventually breaking it and entering the nucleus. **b**, Example of an unsuccessful bead insertion assay. The nuclear envelope is too stiff for the optical trap to enter the nucleus. At t∿5 s, the force is set to 120 pN (top) and the trap pushes the bead against the nucleus. After t∿40 s, the force clamp is turned off. **c**, Example of a successful insertion. Similar to (b), a force clamp of *F*_0_ = 120 pN is initiated at t∿5 s. Around t∿25 s, the nuclear envelope breaks and the trap accelerates into the nucleus. After this, the force clamp is turned off. **d**, Summary for control cells (i), cells incubated with latrunculin A, (ii) and cells with lamin A overexpression (iii). Trajectories have been normalized with respect to the force setpoint as *x*(*t*)*/F*_0_. Colored traces (red: CTR; black: LatA; blue: LMNA + ) indicate successful events in which the bead gets inserted into the nucleus. Gray lines are force compliance curves that didn’t succeed in inserting the bead into the nucleus. **e**, Normalized creep compliance curves over the first 5 s of creep test. Solid lines are medians and shaded areas correspond to the ±25% quantiles. Note, all curves show a fast initial compliance, followed by an inflection indicating an elastic plateau. **f**, Representative data showing the fitting procedure. Yellow line indicates the fit, dotted lines indicate the relevance of the extracted parameters. **g**, Quantification of the force applied to the nucleus during the constant force experiment. Error bars represent the 95% confidence interval of the median. **h-j** Leading parameters extracted from the fit. (**h**) Restoring stiffness of the nuclear envelope; (**i**) resistance to deformation on short time scales and (**j**) resistance to deformation on long time scales. Number of measurements for control N = 15 from n = 4 independent experiments. For LatA N = 13, n = 3 and for LMNA+ N = 20, n = 4. Numbers above the brackets in panel g-j indicate the p-value derived from two-tailed pairwise comparison using a Mann-Whitney U-test on the experimental data points (grey). Violin plots represent the distribution of the bootstrapped mean value (±95% confidence interval) calculated from 5000 virtual experiments, using the experimental data as a sample to describe the population. Source data

Source Data Extended Data Fig. 7 Statistical source data.

**Extended Data Fig. 8:**
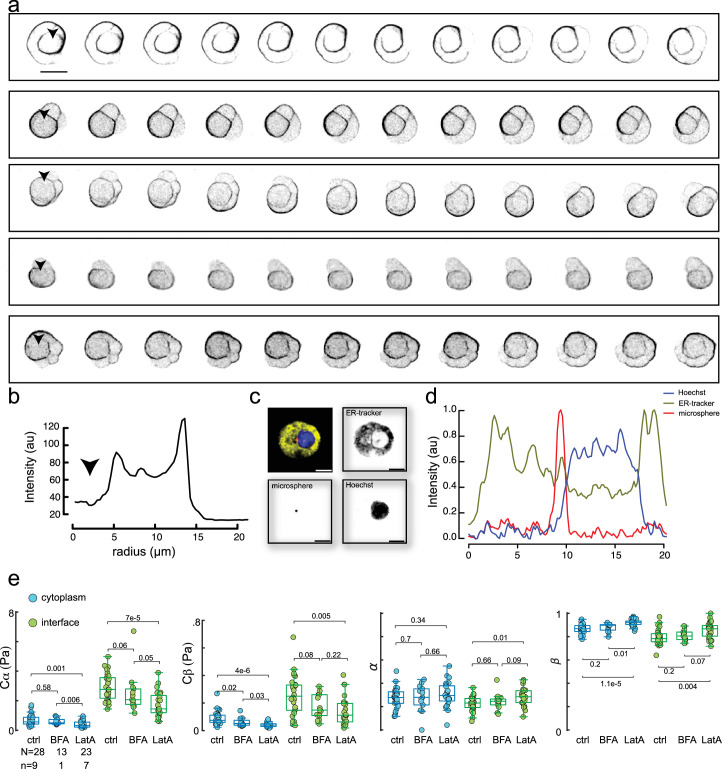
Actin distribution in isolated progenitor cells undergoing blebbing behavior. **a**, Montage of five different isolated mesendoderm progenitor cells (representative for N = 15 cells), showing dynamics of filamentous actin stained with Lifeact-GFP. Arrowhead indicates the location where the nucleus is expected. Note, how the ‘old’ cortex collapses onto the cell center and accumulates around the nucleus. Length of each sequence, t = 120 s. Scalebar=10 µm. Embryos were injected with 50 pg lifeact-GFP to visualize filamentous actin and were isolated in sphere/dome stage according to the methods described in this manuscript. Data set from ref. ^36^. **b**, Radial profile plot of the averaged intensity as a distance from the cell center. Arrowhead points to the nuclear location. Representative for N = 10 cells. **c**, Image of a zebrafish cell (representative for n = 5) stained with ER-tracker to label the endoplasmatic reticulum, Hoechst to label the nucleus to highlight the location of the bead with respect to these organelles. Scale bars=10 *µ*m. **d**, Radial profile plot showing the intensity distribution of the three channels. Note, the microsphere is in contact with the nucleus, without large accumulation of ER in between, as judged from the absence of the green ER signal. **e**, Parameters extracted from the microrheology routine on zebrafish cells treated with 5 *µ*g/mL BrefeldinA (BFA) and LatA for comparison to the data in Fig. 4 and Extended Data Fig. 5. P-values are indicated in the combinations, derived from a two-sided Mann-Whitney U-test. Boxes indicates the central 50% of the data around the median (horizontal line), and the whisker delimit the 10 and 90^th^ percentile. N=number of cells measured over n independent experiments. Source data

Source Data Extended Data Fig. 8 Statistical source data.

**Extended Data Fig. 9:**
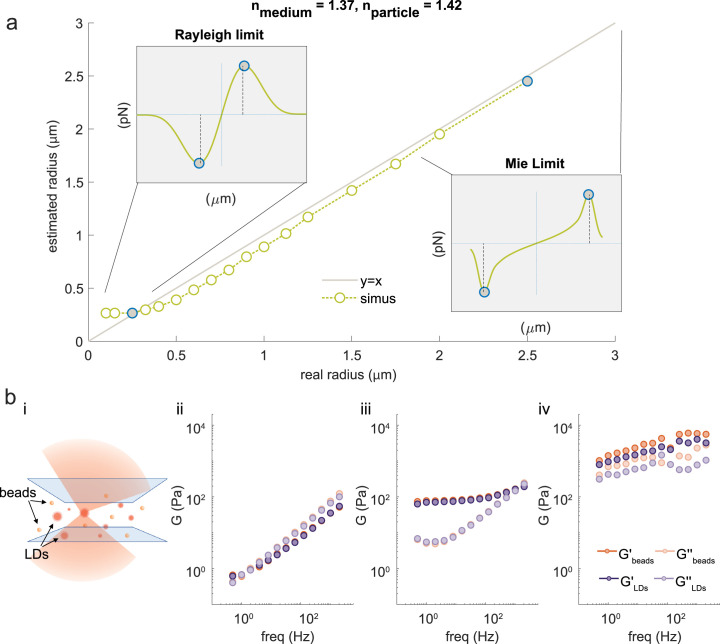
Lipid droplets correctly measure rheological properties up to several kPa. **a**, Estimation of the LD size using simulations with the Optical Tweezers Toolbox^72^. The refractive index of the lipid droplet was considered *n*_LD_ = 1.42 and that of the tissue *n*_worm_ = 1.37. The numerical aperture of the laser trapping beam was approximated to 1 as the entrance pupil of our NA = 1.2 trapping objective is underfilled with a factor of 0.8. We considered radii from *R* = 0.1 µm to *R* = 2.5 µm. From the force profiles, we could measure the peak-to-peak radius, and finally obtained a look-up table from which the *real radius* is obtained from the *estimated radius* from the force scan across the LD. Insets i and ii show examples in the Rayleigh and Mie regimes, respectively. While the radius of probes in the strict Rayleigh regime degenerates and cannot be addressed, the estimated radius tends to the real radius in the Mie regime (geometrical optics). **b**, (i) Schematic of the experiment. Lipid droplets are isolated from *C. elegans* and embedded into a poly-acrylamide gel with varying crosslinker (bis-acrylamide) concentration. (ii-iv) Comparison of the rheological spectrum performed with the lipid droplets and the polystyrene microspheres, embedded into a gel with ii) 2-10 Pa, iii) 10-100 and iv) 100-1000 Pa elastic modulus. Source data

Source Data Extended Data Fig. 9 Statistical source data.

**Extended Data Fig. 10:**
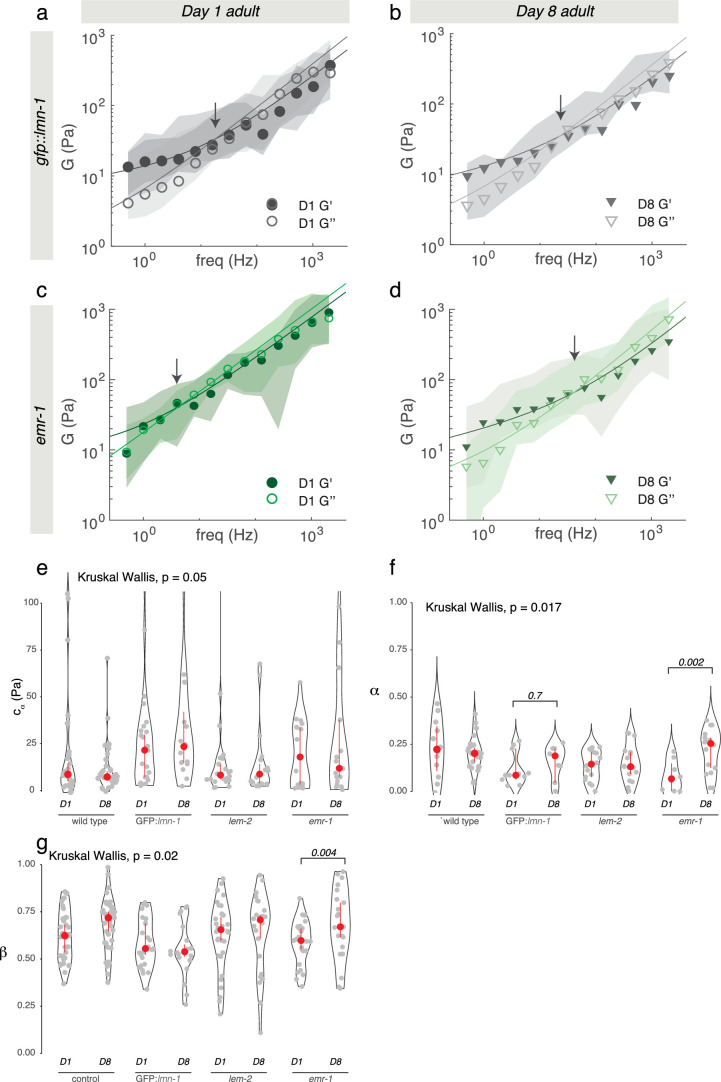
Mutations in nuclear envelope sensitize the cytoplasm of *C. elegans* to age-related changes in viscoelasticity. **a, b**, Rheological spectrum derived from dominant-negative *gfp:lmn-1* expressing animals at (a) day 1 and (b) day 8 adulthood. **c, d**, Rheological spectrum derived from *emr-1* mutant animals at (c) day 1 and (d) day 8 adulthood. The median and ±25% quantiles are represented by lines and shadows, respectively. **e**, Prefactor *C*_*α*_ of the low-frequency component derived from the fit of the fractional Kelvin-Voigt model to the rheological spectrum in Fig. 5e, f and panels a-c above. **f**, Exponent *α* of the low-frequency component derived from the fit of the fractional Kelvin-Voigt model to the frequency spectrum. **g**, Exponent *β* of the high-frequency component derived from the fit of the fractional Kelvin-Voigt model to the frequency spectrum. Red circle indicates median bootstrapped ±95% confidence interval. P-values derived from one-sided Kruskal-Wallis followed by pairwise Dunn test without adjustment. For statistical comparison and p-values see Supplementary Table 2. Number of biological replicates (n = # of worms) and technical replicates (N = # of spectra on different intestinal cells inside the worm). Source data

Source Data Extended Data Fig. 10 Statistical source data.

## Data Availability

All numerical data are available via Zenodo at 10.5281/zenodo.14215139 (ref. ^70^). Source data are provided with this paper.

## Code availability

The code to perform the TimSOM FDE simulation is available via GitLab at https://gitlab.icfo.net/rheo/Tweezers/timsom.
