# Publisher Correction: Measuring age-dependent viscoelasticity of organelles, cells and organisms with time-shared optical tweezer microrheology

**DOI:** 10.1038/s41565-025-01901-8

**Published:** 2025-04-17

**Authors:** Frederic Català-Castro, Santiago Ortiz-Vásquez, Carmen Martínez-Fernández, Fabio Pezzano, Carla Garcia-Cabau, Martín Fernández-Campo, Neus Sanfeliu-Cerdán, Senda Jiménez-Delgado, Xavier Salvatella, Verena Ruprecht, Paolo-Antonio Frigeri, Michael Krieg

**Affiliations:** 1 ICFO—Institut de Ciències Fotòniques, Castelldefels, The Barcelona Institute of Science and Technology, Barcelona, Spain; 2 Center for Genomic Regulation (CRG), The Barcelona Institute of Science and Technology, Barcelona, Spain; 3 Institute for Research in Biomedicine (IRB Barcelona), The Barcelona Institute of Science and Technology, Barcelona, Spain; 4 ICREA, Barcelona, Spain; 5 Universitat Pompeu Fabra (UPF), Barcelona, Spain; 6 Impetux Optics, Barcelona, Spain

**Keywords:** Biomaterials, Biosensors, Biological physics, Characterization and analytical techniques, Biomedical engineering

Correction to: *Nature Nanotechnology* 10.1038/s41565-024-01830-y, published online 2 January 2025.

In the version of this article initially published, Frederic Català-Castro, Santiago Ortiz-Vásquez and Carmen Martínez-Fernández were not listed as equal contributors to the work, as is now amended in the HTML and PDF versions of the article.

