# Supplementary material for: Measuring age-dependent viscoelasticity of organelles, cells and organisms with time-shared optical tweezer microrheology
Source: Nat Nanotechnol. 2025 Jan 2;20(3):411–20. doi: 10.1038/s41565-024-01830-y (PMC11919717; doi:10.1038/s41565-024-01830-y)
Supplement: Supplementary file 2 — Reporting Summary [file 41565_2024_1830_MOESM2_ESM.pdf]

nature portfolio

Corresponding author(s): Michael Krieg

Last updated by author(s): Oct 11, 2024

Reporting Summary

Nature Portfolio wishes to improve the reproducibility of the work that we publish. This form provides structure for consistency and transparency in reporting. For further information on Nature Portfolio policies, see our Editorial Policies and the Editorial Policy Checklist.

Statistics

For all statistical analyses, confirm that the following items are present in the figure legend, table legend, main text, or Methods section.

n/a	Confirmed	
☐	☒	The exact sample size (n) for each experimental group/condition, given as a discrete number and unit of measurement
☐	☒	A statement on whether measurements were taken from distinct samples or whether the same sample was measured repeatedly
☐	☒	The statistical test(s) used AND whether they are one- or two-sided<br>*Only common tests should be described solely by name; describe more complex techniques in the Methods section.*
☒	☐	A description of all covariates tested
☐	☒	A description of any assumptions or corrections, such as tests of normality and adjustment for multiple comparisons
☐	☒	A full description of the statistical parameters including central tendency (e.g. means) or other basic estimates (e.g. regression coefficient) AND variation (e.g. standard deviation) or associated estimates of uncertainty (e.g. confidence intervals)
☐	☒	For null hypothesis testing, the test statistic (e.g. F, t, r) with confidence intervals, effect sizes, degrees of freedom and P value noted<br>*Give P values as exact values whenever suitable.*
☒	☐	For Bayesian analysis, information on the choice of priors and Markov chain Monte Carlo settings
☒	☐	For hierarchical and complex designs, identification of the appropriate level for tests and full reporting of outcomes
☒	☐	Estimates of effect sizes (e.g. Cohen's d, Pearson's r), indicating how they were calculated

Our web collection on statistics for biologists contains articles on many of the points above.

Software and code

Policy information about availability of computer code

Data collection	LightAce 1.6.2.0 SDK for acquiring optical tweezer force spectroscopy and for active microrheology, Fusion Andor 2.1.0.80 for imaging (as described in SI Text 6)
Data analysis	RheoAnalysis 1.7.2 for analysis of active microrheology data, Matlab and Python own scripts for simulations, FIJI ImageJ I.54f for image analysis; custom software code to run the FED simulation is found in https://gitlab.icfo.net/rheo/Tweezers/timsom

For manuscripts utilizing custom algorithms or software that are central to the research but not yet described in published literature, software must be made available to editors and reviewers. We strongly encourage code deposition in a community repository (e.g. GitHub). See the Nature Portfolio guidelines for submitting code & software for further information.

Data

Policy information about availability of data

All manuscripts must include a data availability statement. This statement should provide the following information, where applicable:

- Accession codes, unique identifiers, or web links for publicly available datasets
- A description of any restrictions on data availability
- For clinical datasets or third party data, please ensure that the statement adheres to our policy

All source numerical data is appended as supplementary data file to the current manuscript. In addition it can be found on zenodo under 10.5281/zenodo.13910465

Research involving human participants, their data, or biological material

Policy information about studies with human participants or human data. See also policy information about sex, gender (identity/presentation), and sexual orientation and race, ethnicity and racism.

Reporting on sex and gender	does not apply
Reporting on race, ethnicity, or other socially relevant groupings	does not apply
Population characteristics	does not apply
Recruitment	does not apply
Ethics oversight	does not apply

Note that full information on the approval of the study protocol must also be provided in the manuscript.

Field-specific reporting

Please select the one below that is the best fit for your research. If you are not sure, read the appropriate sections before making your selection.

☒ Life sciences ☐ Behavioural & social sciences ☐ Ecological, evolutionary & environmental sciences

For a reference copy of the document with all sections, see nature.com/documents/nr-reporting-summary-flat.pdf

Life sciences study design

All studies must disclose on these points even when the disclosure is negative.

Sample size	All samples sizes are indicated in the figures or in the figure legends. No method to predetermine sample size was used. Sample sizes are contingent with the standard of the field.
Data exclusions	No data was excluded unless otherwise described in the manuscript.
Replication	All measurement are replicable and all attempts are described in the figures and their legends.
Randomization	No randomization applied to the study design, e.g the frequency sweep was performed from high to low frequencies. When tested directly, no history effect was observed, thus, randomization was not necessary for the repeatability of the results.
Blinding	Unless otherwise specified, no blinding was applied. Data in Fig. 5 was analyzed blind to genotype.

Reporting for specific materials, systems and methods

We require information from authors about some types of materials, experimental systems and methods used in many studies. Here, indicate whether each material, system or method listed is relevant to your study. If you are not sure if a list item applies to your research, read the appropriate section before selecting a response.

Materials & experimental systems

n/a	Involved in the study	
☒	☐	Antibodies
☒	☐	Eukaryotic cell lines
☒	☐	Palaeontology and archaeology
☐	☒	Animals and other organisms
☒	☐	Clinical data
☒	☐	Dual use research of concern
☒	☐	Plants

Methods

n/a	Involved in the study	
☒	☐	ChIP-seq
☒	☐	Flow cytometry
☒	☐	MRI-based neuroimaging

Animals and other research organisms

Policy information about studies involving animals; ARRIVE guidelines recommended for reporting animal research, and Sex and Gender in Research

Laboratory animals	Dania rerio (AB lab strain) and Caenorhabditis elegans (N2 wildtype and derivatives[Table S7])

Wild animals	does not apply
Reporting on sex	Young adult hermaphrodite nematode animals have been used throughout the study, in concordance with the standard in the field.
Field-collected samples	not applicable
Ethics oversight	All zebrafish protocols used have been approved by the Institutional Animal Care and Use Ethic Committee (PRBB–IACUEC) and implemented according to national and European regulations. All experiments were carried out in accordance with the principles of the 3Rs (replacement, reduction, and refinement). C. elegans is a nematode and thus exempt from ethical approval and regulations.

Note that full information on the approval of the study protocol must also be provided in the manuscript.

Plants

Seed stocks	does not apply
Novel plant genotypes	does not apply
Authentication	does not apply
